# Preparation for mitosis requires gradual CDK1 activation

**DOI:** 10.1016/j.isci.2025.112292

**Published:** 2025-03-25

**Authors:** Karen Akopyan, Zhiyu Hao, Arne Lindqvist

**Affiliations:** 1 Department of Cell and Molecular Biology, Karolinska Institutet, Biomedicum A7, 171 77 Stockholm, Sweden

**Keywords:** Cell biology, Functional aspects of cell biology, Mathematical biosciences

## Abstract

G2 phase is considered as a time in which cells prepare for the large structural changes in the following mitosis. Starting at completion of DNA replication, CDK1 and PLK1 kinase activities gradually increase throughout G2 phase until reaching levels that initiate mitosis. Here, we use a combination of experiments and a data-driven mathematical model to study the connection between DNA replication and mitosis. We find that gradual activation of mitotic kinases ensures CDK1-dependent transcription of factors required for mitosis. In addition, we find that gradual activation of CDK1 coordinates CDK1 and PLK1 activation. Conversely, shortening G2 phase by WEE1 inhibition leads to mitotic delays, which can be partially rescued by expression of constitutively active PLK1. Our results show a function for slow mitotic kinase activation through G2 phase and suggest a mechanism for how the timing of mitotic entry is linked to preparation for mitosis.

## Highlights

• A data-driven ODE model of interphase
• CDK1-PLK1-transcription feedback ensures slow and timely kinase activation
• Slow CDK1 activation in G2 coordinates expression and activation of mitotic proteins
• Duration of forced mitosis after WEE1i in G2 partially rescued by active Plk1

## Graphical abstract

**Figure:**
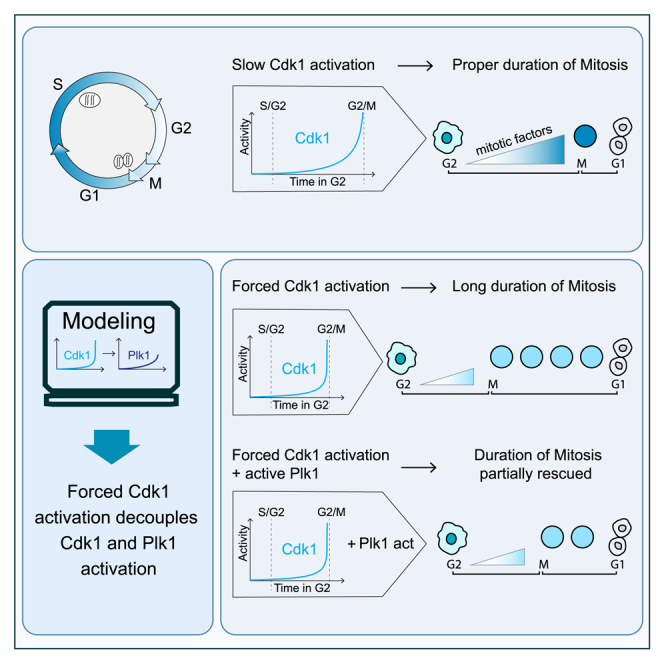

## Introduction

Cell division forms the base for multiplication of cells. To preserve genome integrity, cell division should only occur once all DNA has been replicated. For most mammalian cells, there is a long pause between DNA replication and cell division that is referred to as G2 phase. Why G2 phase frequently takes hours is not clear. Initial observations led to the idea that G2 phase was required for protein synthesis,^1^ including the synthesis of Cyclins that help trigger mitosis.^2^ However, more recent work showed that protein synthesis in G2 phase is not strictly required for mitotic entry.^3^ Moreover, inhibition of Wee1 can force mitotic entry even before a cell has entered G2 phase, raising the question why a long G2 phase exists.^4^

Mitosis is triggered by Cdk1 in complex with Cyclin A or Cyclin B. This Cdk1 activity is suppressed by ongoing DNA replication, ensuring that mitosis is not triggered during S phase.^5^^,^^6^ Once DNA replication is complete, the suppression is lifted, and initial Cdk1 activity can be detected at the S/G2 border. This low-level Cdk1 activity gradually increases throughout G2 phase, until increasing in an exponential fashion, which eventually triggers mitosis.^7^

The increase of Cdk1 activity throughout G2 phase can be attributed to the presence of multiple feedback loops.^8^ First, Cdk1 activity stimulates transcription of factors that will further increase Cdk1 activation.^6^^,^^9^^,^^10^^,^^11^ Second, Cdk1 activity posttranslationally modifies regulators of Cdk1, thereby increasing Cdk1 activity. Whereas some feedback loops are direct, such as activation of Cdc25s,^12^ other involve multiple components such as regulation of Wee1 and Cdc25 by Polo-like kinase 1 (Plk1),^13^^,^^14^ which in turn can be activated by Cdk1-mediated phosphorylation of the Aurora A cofactor Bora^15^^,^^16^^,^^17^ or Cdk1-mediated phosphorylation of WW domain-containing adapter protein with coiled-coil (WAC).^18^

Due to the feedback loops, Cdk1 has been shown to function as a bistable switch.^19^^,^^20^^,^^21^^,^^22^ Importantly, the presence of bistability shows that, once initiated, Cdk1 activation will progress until reaching a high activity steady state but does not say how long it will take for the switch to flip. Apparently, for most human cells, the duration is several hours, but what possible benefits could be for a long G2 phase are unknown.

Here, we have investigated the feedback that stimulates Cdk1 activation. By combining experiments with a data-driven mathematical model, we find that slow Cdk1 activation through G2 phase couples the timing of mitotic entry with activation of Plk1 and expression of proteins required for mitosis.

## Results

### Cdk1 regulates Cyclin B1 accumulation during G2 phase

**Figure 1:**
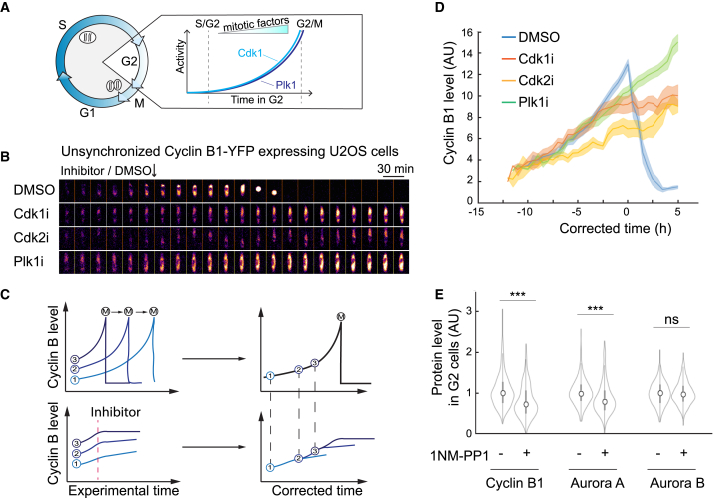
Cdk1 regulates Cyclin B accumulation in G2 phase (A) Schematic. Cdk1 and Plk1 activities that induce mitosis are detected at the S/G2 border and increase gradually throughout G2 phase. (B) Example of unsynchronized U2OS Cyclin B1-YFP cells growing on micropatterns. Lighter colors indicate higher Cyclin B1-YFP fluorescence. Arrows indicate addition of DMSO or inhibitors. Time lapse 30 min. Scale bar 20 μm. (C) Schematic of the *in silico* synchronization setup. DMSO-treated cells are synchronized in mitosis (top). The resulting Cyclin B1-YFP fluorescence curve (top right) is used to fit Cyclin B1-YFP fluorescence of individual cells before kinase inhibitor treatment (bottom). (D) Quantification of Cyclin B1-YFP fluorescence of cells growing on micropatterns, treated with DMSO or indicated kinase inhibitors after *in silico* synchronization as in (B) and (C). Graph shows average and standard error of Cyclin B1-YFP fluorescence (At least 15 cells per condition are showed. Data are representative of 4 additional independent experiments, except for Plk1 inhibitor that is present in 2 additional independent experiments). Please note that only Cyclin B1-YFP fluorescence after kinase inhibitor addition is plotted. (E) Quantification of Cyclin B1, Aurora A, and Aurora B immunofluorescence in 4N U2OS Cdk1as cells after 2 h treatment with 1NMPP1. At least 420 cells per condition are showed. Data are representative of 3 independent experiments. ∗∗∗*p* < 0.001, using Student’s t test. Interquartile range and median values are indicated within violin plots.

We sought to test the importance of gradual activation of mitotic kinases in G2 phase (Figure 1A). We first considered the hypothesis that mitotic kinase activation contributes to production of mitotic proteins. We reasoned that Cyclin B1 could function as a proxy for a regulated mitotic protein, as its expression is undetectable in G1 phase, remains limited during early S phase, and increases dramatically during G2 phase.^23^ We therefore utilized a cell line in which we have targeted endogenous Cyclin B1 with yellow fluorescent protein (YFP).^7^ We monitored single U2OS Cyclin B1-YFP cells growing on micropatterns to decrease variability due to cell-extrinsic factors and to increase quantification accuracy of Cyclin B1-YFP levels over time^7^ (Figure 1B).

We next added small-molecule kinase inhibitors to unsynchronized cells and quantified Cyclin B1-YFP levels over time. We chose to test inhibitors of Cdk1 (RO3306), Cdk2 (NU6140), and Plk1 (BI2536), three kinases that are central for cell-cycle progression and active throughout G2 phase (Figure 1B). To pinpoint if and when an inhibitor impacted Cyclin B1-YFP accumulation, we devised an *in silico* synchronization scheme. We first aligned control cells to mitotic entry, creating a trendline of Cyclin B1-YFP accumulation over time (Figure 1C, top panels; Figure 1D, DMSO). Using the Cyclin B1-YFP levels before inhibitor addition, we next aligned all cells to the trendline, thereby assigning an approximate cell-cycle position for each cell (Figure 1C, lower panel). We then plotted Cyclin B1-YFP accumulation after addition of each inhibitor and compared it to the trendline of control cells. Importantly, this approach is designed to identify if and when in the cell cycle Cyclin B1-YFP accumulation is initially affected by inhibitor addition but may not be quantitative for the magnitude of changes after an effect is observed.

Addition of NU6140 led to decreased Cyclin B1-YFP accumulation more than 6 h before mitosis, whereas addition of RO3306 or BI2536 led to decreased Cyclin B1-YFP accumulation approximately 3 h before mitosis (Figure 1D). The data are in agreement with the activity profiles of the targets of the inhibitors, as Cdk2 activity is progressively increasing through S phase, and Cdk1 and Plk1 are progressively increasing through G2 phase.^7^^,^^24^ Our results would be consistent with a model in which Cdk2 ensures a baseline of Cyclin B1 accumulation already from S phase, whereas Cdk1 activity and to some extent Plk1 activity enable the increase in Cyclin B1 levels during G2 phase.

To test whether Cdk1 activity drives the increase of Cyclin B1 levels in G2 phase, we used immunofluorescence to quantify endogenous Cyclin B1 levels in an unsynchronized population of U2OS Cdk1as cells. In these cells, Cdk1 is modified to allow selective inhibition using the bulky ATP analog 1NMPP1.^21^ Focusing on cells with a 4N DNA content, we note that cells treated for 2 h with 1NMPP1 show reduced staining for Cyclin B1 (Figure 1E). We conclude that Cdk1 activity increases Cyclin B1 levels in G2 phase.

### Cdk1 stimulates synthesis of mitotic factors and of proteins that boost its own activity

**Figure 2:**
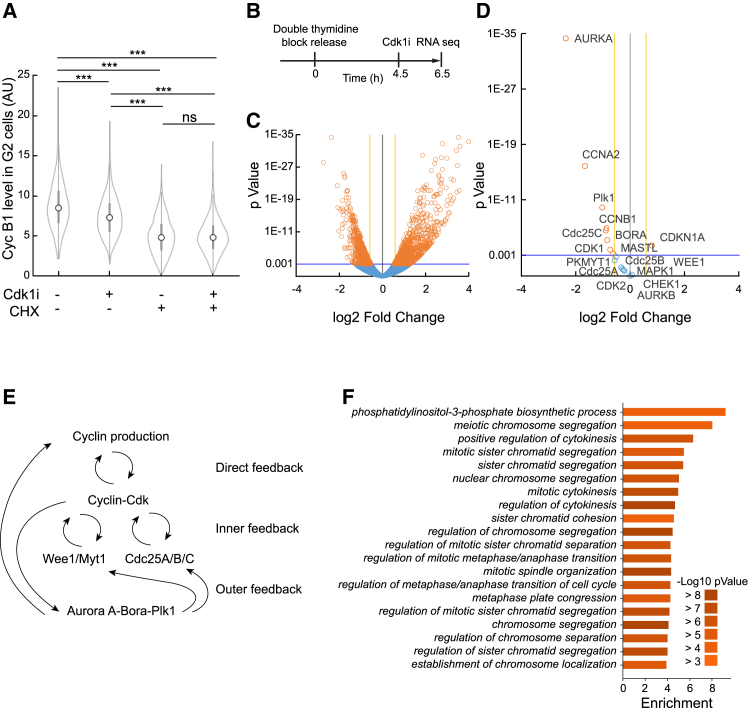
Cdk1 regulates transcription of mitotic factors (A) Quantification of Cyclin B1 immunofluorescence in 4N U2OS cells treated with Cdk1 inhibitor (RO3306), cycloheximide, or both for 2 h. The G2 population was separated *in silico* based on DAPI. At least 520 cells per condition are showed. Data are representative of 2 independent experiments; ∗∗∗*p* < 0.001, using ANOVA. Interquartile range and median values are indicated within violin plots. (B) Schematic of the setup for RNA sequencing. HeLa cells were released after double thymidine synchronization. After 4.5 h Cdk1 inhibitor (RO3306) or DMSO was added. After 2 h cells were harvested for RNA sequencing analysis. (C and D) Volcano plots show log2 fold change between treated (RO3306) and non-treated (DMSO) normalized gene expressions (x axis), plotted versus the *p* value (y axis). Orange circles represent differentially expressed genes, and blue circles represent genes with similar expression (data from 3 independent experiments). (E) Schematic containing a selection of key components involved in direct, inner, and outer feedback regulating Cdk activity. (F) Gene Ontology and *p* values based on (C).

The increase in Cyclin B1 levels late in the cell cycle largely stems from upregulated transcription, and Cdk activity has been implicated in increasing transcription of Cyclin B1, in particular, by modification of FoxM1.^6^^,^^9^^,^^10^^,^^25^ To test if Cdk1 inhibition affects Cyclin B1 production, we treated unsynchronized U2OS cells with the translation inhibitor cycloheximide (CHX) and analyzed Cyclin B1 content in G2 cells by quantitative immunofluorescence. Whereas RO3306 addition for 2 h reduced Cyclin B1 levels over control cells, treatment with CHX or CHX together with RO3306 showed a similar decline in Cyclin B1 levels (Figure 2A), suggesting that RO3306 addition can affect production of Cyclin B1.

To test if RO3306 addition affected transcription of Cyclin B1, we next synchronized HeLa cells by release from an S-phase arrest, treated with RO3306, and sequenced poly A-enriched RNA (Figure 2B). In line with the previous results, we find Cyclin B1 (CCNB1) mRNA levels to be reduced after RO3306 addition in G2 phase. Notably, mRNA levels of other cell-cycle regulators as Aurora A (AURKA), Plk1, Greatwall (MASTL), and Cyclin A2 (CCNA2) were also reduced (Figures 2C and 2D). Synchronization typically is not complete and may lead to effects that are not present in unperturbed cells. Similarly, RO3306 treatment may show off-target effects and in a Cdk1-independent manner affect cell-cycle progression.^26^^,^^27^ We therefore quantified the expression of key cell-cycle regulators after 1NMPP1 treatment in single unsynchronized G2 phase U2OS Cdk1as cells using quantitative immunofluorescence. We verified that Aurora A expression was affected by addition of 1NMPP1 to Cdk1as cells in G2 phase, whereas the related mitotic kinase Aurora B was not affected at mRNA level after RO3306 addition or at protein level after 1NMPP1 addition (Figure 1, Figure 2, 2D, and S1A). Similar trends were seen for the cell-cycle regulators Cyclin A2, Plk1, Greatwall, and p21 (Figure S1B). Thus, at least for these regulators, two different approaches of Cdk1 inhibition in synchronized and unsynchronized G2 phase gave comparable results.

Cdk activity is increasing in an exponential fashion during G2 phase, which is attributed to the presence of multiple feedback loops. These feedback loops can be divided into direct feedback on cyclin-Cdk complex levels, inner feedback loops that directly activate Cyclin-Cdk, and outer feedback loops that indirectly activate Cyclin-Cdk^8^ (Figure 2E). It is unclear how much transcriptional regulation contributes to the various feedback systems. We therefore searched for changes in mRNA levels that would be expected to impact Cdk activity. Apart from Cyclin B1, Cyclin A2 and Cdk1 are among the mRNAs that decreased upon RO3306 addition in G2 phase (Figure 2D). This suggests that Cdk1 activation in G2 phase stimulates the presence of more Cyclin-Cdk1 complexes. However, with the exception of Cdc25C, mRNA levels for the direct Cyclin-Cdk regulators Cdc25A, Cdc25B, Wee1, and Myt1 were unaffected by RO3306 addition, suggesting that transcriptional regulation is limited for the inner feedback loops that activate Cdk1 through posttranslational modifications. Interestingly, mRNA for Aurora A, Bora, and Plk1, which indirectly can amplify Cdk1 activity, was reduced after RO3306 addition in G2 phase. Thus, transcriptional regulation seems to exist within the outer feedback loops for Cdk1 activation.

Except Cyclin B1 and Aurora A, the mRNA levels of many genes are affected by RO3306 treatment in G2 phase (Figures 2C and 2D). Using Gene Ontology (GO) analysis, we find enrichment for various processes that are important for mitosis, including mitotic spindle formation, regulation of chromosome segregation, and cytokinesis. Although we cannot exclude that expression differences of individual genes were influenced by synchronization or unspecific binding by RO3306, this suggests that Cdk1 activity in G2 phase can stimulate the production of multiple proteins that regulate mitosis (Figures 2F and S1C).

In conclusion, our data would be consistent with a model in which Cdk1 activity in G2 phase stimulates both the transcription of many proteins with mitotic functions and that of proteins that stimulate further Cdk1 activation. The transcriptional component seems to be strong in particular for the kinases Aurora A and Plk1 that in addition to stimulate Cdk1 activity also are key regulators of mitotic processes.

### Model

**Figure 3:**
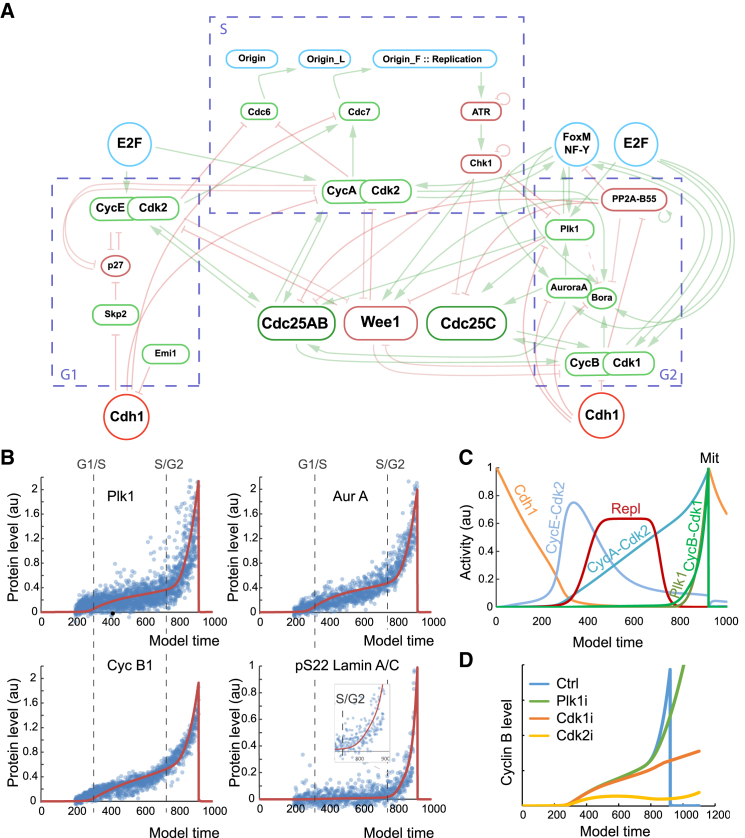
Mathematical model of the cell cycle (A) Schematic representation of the mathematical model. (B) Model prediction (red line) of protein level dynamics after parameter estimation based on quantitative immunofluorescence of indicated proteins in U2OS cells from Akopyan et al.^7^ (blue dots). Please note that experimental data are only used until mitotic entry. The decrease of protein levels in mitosis is added to the model to mark mitosis upon full activation of Cdk1. (C) Model estimation of selected cell-cycle activities. Model time refers to number of calculation steps with a fixed duration. (D) Model prediction of Cyclin B level dynamics after inhibition of Cdk1, Cdk2, or Plk1.

To better understand the implications of Cdk-mediated regulatory feedback, we decided to use a simulation approach based on experimental data. We sought to create a model that balances overparameterization when fitting to data by only including a limited set of main players that regulate Cdk activity. At the same time, we sought to include an updated framework of cell-cycle regulation concerning the large part played by DNA replication on regulating interphase^28^ (Figure 3A).

The ordinary differential equation (ODE) model is based on the combined action of feedforward and feedback loops. In S phase, Cdk2 complexes inactivate origin licensing (in the model for simplicity simulated as Cdc6) and activate origin firing (simulated as Cdc7), which initiates DNA replication. While DNA replication is ongoing, it inhibits the activities of main cell-cycle kinases (simulated as ATR and Chk1). Cdk2 also participates in production and activation of mitotic inducers and thus together with DNA replication takes part in a feedforward loop that connects DNA replication and mitosis. When DNA replication is completed in G2 phase, various feedback loops in the mitotic entry network ensure that Plk1 and Cdk1 activities will rise and bring the cell to mitosis. The transcriptional feedback through Cdk1 based on Figure 2 is simulated as FoxM1. For simplicity, both Cyclin A-Cdk1 and Cyclin B-Cdk1 are simulated as Cyclin B-Cdk1. To start the cell cycle, we used a model of the G1/S transition from Novák and coauthors, which has been fitted to experimental data.^29^ For a detailed description of the model, see Data S1.

After an initial manual selection of parameters, we used experimental data from our previous work^7^ to fit model parameters to reflect changes in protein levels of Cyclin B1, Plk1, and Aurora A through interphase until mitotic entry. Similarly, we fitted model parameters to reflect Cdk1 activity based on the level of phosphorylated Lamin A/C (Figure 3B). The estimations of time and cell-cycle transitions in the experimental data are based on ordering of cells and comparison to data from time-lapse microscopy^7^ (Figure S2A) and are compared to model time indicating representation of time within the model (Data S1). The model containing updated parameters is in agreement with main trends of order and shape of main cell-cycle regulators and their activities (Figure 3C).

To test the accuracy of the model, we decided to reproduce the situation described in Figure 1D. We therefore simulated inhibition of Cdk1, Cdk2, or Plk1 and monitored the resulting Cyclin B level. The simulation recapitulated the trends of the experimental data, indicating that the model can describe main aspects of how Cdk1, Cdk2, and Plk1 are linked to Cyclin B production (Figures 1D and 3D).

We next sought to test the predictive power of the model for a variable that was not fitted to experimental data. A key aspect of the model is that DNA replication functions as a signaling component that limits G2-specific activities and thereby participates in a feedforward loop that coordinates S phase and mitosis.^28^ Simulating a reduction in DNA replication origin licensing by reducing the levels of Cdc6, we see that DNA replication occurs at a lower speed and, consequently, takes longer (Figure S2B). As the levels of Cdc6 decrease, the inhibition from DNA replication on mitotic kinases is not sustained until completion of DNA replication, and the cell eventually enters mitosis without replicating all DNA (Figure S2C). Importantly, whereas the cell cycle is prolonged at intermediate levels of Cdc6, very low levels of Cdc6 lead to premature activation of mitotic kinases and, consequently, to a shorter cell cycle (Figure S2D). These data are in agreement with observations using degron-tagged Cdc6 in cells,^5^ indicating that the model can recapitulate the basic logic of how DNA replication coordinates activities through S and G2 phases.

### Mitotic duration after Wee1 inhibition depends on when in G2 phase Wee1 inhibitors are added

**Figure 4:**
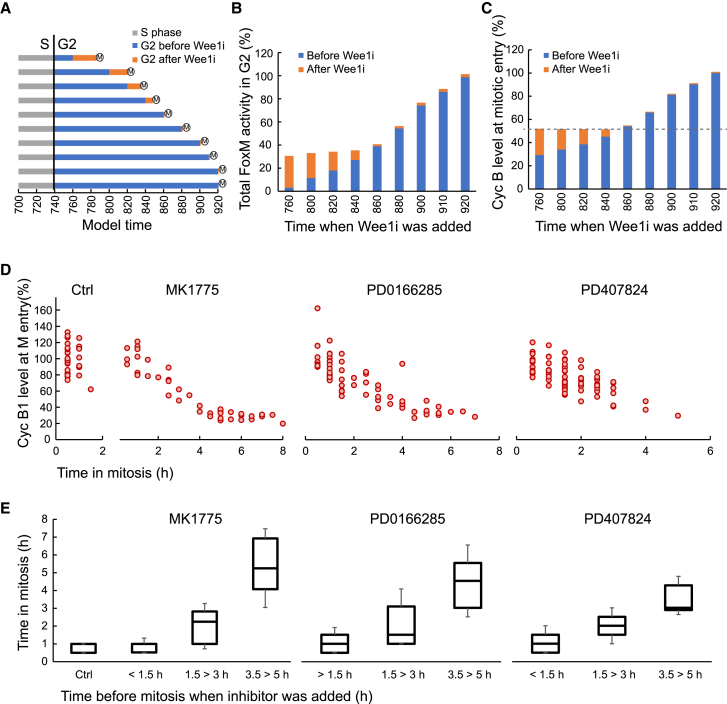
Mitotic duration after Wee1 inhibition depends on when in G2 phase Wee1 inhibitors are added (A) Model prediction of G2 duration after Wee1 inhibition at different time points in G2 phase. Time when Wee1i added same as in (B) and (C). G2 phase starts approximately at model time 740, and 20 model time corresponds approximately to 30 min. How activities change relative to model time is visible in Figure 3. (B) Model prediction of accumulated FoxM activity at mitotic entry after Wee1 inhibition at different time points in G2 phase. 100% denotes mitotic levels in absence of Wee1 inhibition. (C) Model prediction of Cyclin B level at mitotic entry after Wee1 inhibition at different time points in G2 phase. 100% denotes mitotic levels in absence of Wee1 inhibition. Striped line indicates apparent minimum Cyclin B levels at mitotic entry. (D and E) U2OS Cyclin B1-YFP cells were monitored by time-lapse microscopy upon addition of Wee1 inhibitors. Three different inhibitors were used: MK1775 (1 μM), PD0166285 (1 μM), and PD407824 (5 μM). (D) Duration of mitosis (x axis) is plotted versus Cyclin B1-YFP level at mitotic entry (y axis). 100% denotes median mitotic levels in absence of Wee1 inhibition. (E) Duration of mitosis (y axis) is plotted versus estimated time before mitosis should Wee1 inhibitors have not been added (x axis). The estimate is based on Cyclin B1-YFP accumulation of control cells in the same experiment (Figures S3E and S3F). 31–70 cells per condition are showed. The data are representative of 3 independent experiments.

Having established that the model can recapitulate main aspects of how Cdk1, Cdk2, and Plk1 activities and associated protein levels rise through S and G2 phase, we next focused on the importance of Cdk1-dependent feedback. We therefore removed Wee1 activity *in silico* at various points in G2 phase. Not surprisingly, the model rapidly gained full Cdk1 activity and entered mitosis (Figure 4A). When Wee1 activity was removed in early G2 phase, there was a small delay before mitosis, in which G2 transcription was active while Cdk1 activity was rising (Figures 4A and 4B). This delay ensured that, when Wee1 activity was removed, mitosis only occurred once a threshold level of Cyclin B1 was reached, which corresponded to the Cyclin B level present in mid-G2 phase (Figure 4C).

We next tested this prediction experimentally by addition of three different Wee1 inhibitors (MK1775, PD0166285, and PD407824) to cells in which Cyclin B1 is tagged to YFP in the endogenous locus. As expected, forcing premature mitosis by Wee1 inhibitor addition led to the presence of mitotic cells with reduced levels of Cyclin B1-YFP (Figures 4D and S3A). In accordance with simulation, all mitotic cells contained a basal expression of Cyclin B1-YFP. However, the detected minimal level of Cyclin B1-YFP in mitosis is lower than minimal Cyclin B during simulation, possibly reflecting that both Cyclin A2-Cdk1 and Cyclin B1-Cdk1 are grouped as Cyclin B-Cdk1 during simulation (Figures 4C and 4D).

To study the consequences of Wee1 inhibition, we next monitored mitotic progression of single Wee1 inhibitor-treated cells. We note that there is a large variability in mitotic durations after Wee1i addition in G2 phase. The delays occur both before and after congression of chromosomes to the metaphase plate (Figure S4). Apart from the delay, we did not detect an apparent increase in mitotic defects such as lagging chromosomes during anaphase.

We find that cells that passed mitosis with similar timings as control cells also contained Cyclin B-YFP levels similar to control cells. In contrast, cells that showed a prolonged mitosis contained lower levels of Cyclin B-YFP. The level of Cyclin B-YFP before Wee1 inhibitor addition was inversely related to the duration of mitosis (Figures 4D and S3A).

Using an *in silico* approach, we estimated how far into G2 phase each cell was at the time of Wee1 inhibition. Similar to in Figure 1C, the estimate is based on relating Cyclin B-YFP levels of each cell to an average trendline of how Cyclin B-YFP levels increase throughout G2 phase. We find that mitotic duration is inversely related to when in G2 phase Wee1 inhibitors are added (Figure 4E and S3B–S3F).

Thus, our findings indicate that forced Cdk activation in G2 phase results in mitotic entry before all mitotic proteins have accumulated to levels observed in unperturbed cells. We find that Wee1 inhibition is not toxic for mitotic progression if added in late G2 phase. However, if the inhibitor is added earlier in G2 phase, it leads to mitotic delays. Taken together, this indicates that G2 progression and intact Cdk regulation during G2 phase are important for mitotic progression.

### Wee1 inhibition in G2 phase leads to a de-coupling of Cdk1 and Plk1 activities

**Figure 5:**
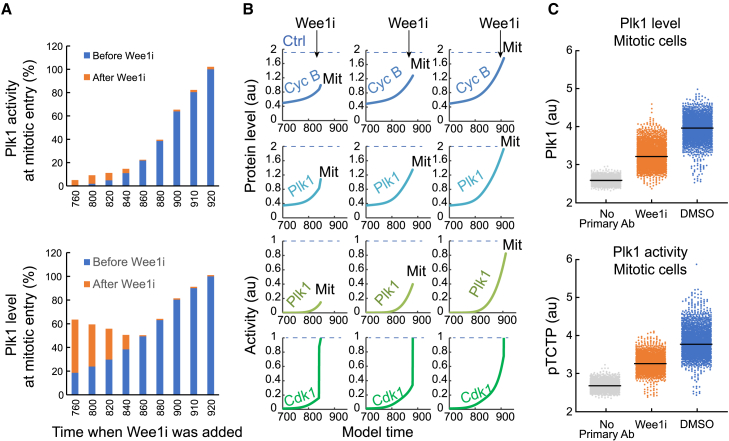
Wee1 inhibition in G2 phase leads to a de-coupling of Cdk1 and Plk1 activities (A) Model prediction of Plk1 activity at mitotic entry after Wee1 inhibition at different time points in G2 phase. 100% denotes mitotic levels in absence of Wee1 inhibition. G2 phase starts approximately at model time 740, and 20 model time corresponds approximately to 30 min. How activities change relative to model time is visible in Figure 3. (B) Model prediction of Cyclin B level, Plk1 level, Plk1 activity, and Cdk1 activity after inhibition of Wee1 at different time points in G2 phase. The dotted line in each graph represents the levels at mitotic entry when Wee1 is not artificially inhibited. Mit indicates mitosis. (C) Flow cytometry analysis of Plk1 levels and Plk1-mediated phosphorylation of TCTP (pTCTP) in mitotic U2OS cells. STLC (10 μM), to block cells in mitosis, was added with or without Wee1i for 2 h before harvest. Graph shows mitotic cells, gated as in Figure S5A. Wee1i, cells treated with the Wee1 inhibitor MK1775 (1 μM) together with STLC; DMSO, cells treated with DMSO together with STLC. At least 2,000 mitotic cells were quantified per condition in two independent experiments.

Apart from production of mitotic proteins, Cdk-dependent feedback involves activation of the mitotic kinase Plk1 that is crucial for mitotic progression.^30^ We therefore simulated how Plk1 levels and activity would be affected by removal of Wee1 activity at various points in G2 phase. In the model, we find that Plk1 activation depends heavily on gradual activation of Cdk1 (Figure 5A). According to simulations, if Wee1i is added early in G2 phase, the cell rapidly develops high Cdk1 activity, intermediate levels of Cyclin B and Plk1, and low Plk1 activity. In contrast, if Wee1i is added late in G2 phase, this affect is less apparent as a large part of both Cdk1-dependent transcription and Plk1 activation already occurred earlier in G2 phase (Figure 5B).

To test this prediction experimentally, we added Wee1i to U2OS cells together with S-trityl-L-cysteine (STLC) to trap cells in mitosis. Using flow cytometry, we find that addition of Wee1i resulted in a decrease of both Plk1 levels and Plk1-mediated phosphorylation of translationally controlled tumor protein (TCTP) in mitotic cells (Figure 5C and S5A). Thus, Wee1i addition in G2 phase influences both Plk1 level and activity when cells enter mitosis.

In the model, removal of Wee1 activity in G2 phase leads to high Cdk1 activity, despite low Plk1 activity (Figure 5B). As high Cdk1 activity in the model defines mitotic entry, we sought to test whether Plk1 activity is necessary for mitotic entry after Wee1i addition. We therefore monitored duration of G2 phase in unperturbed U2OS cells expressing a chromobody to proliferating cell nuclear antigen (PCNA) by time-lapse microscopy. As expected, addition of Plk1i in G2 phase led to a delay before mitotic entry.^13^ However, when Plk1i was added together with Wee1i, mid or late G2 phase cells rapidly entered mitosis. In contrast, a delay in G2 phase was noticed when Wee1i and Plk1i were added very close to the S/G2 transition (Figure S5B). Thus, our data indicate that, once a cell has initiated Cdk1 and Plk1 activities at the S/G2 transition, Plk1 activity is not required for mitotic entry when Wee1 activity is perturbed. However, the possibility remains that, in the absence of Wee1 activity, Plk1 activity is needed to initiate Cdk1 activation early in G2 phase.

### Decoupled Cdk1 and Plk1 activities contribute to prolonged mitosis after Wee1 inhibition

**Figure 6:**
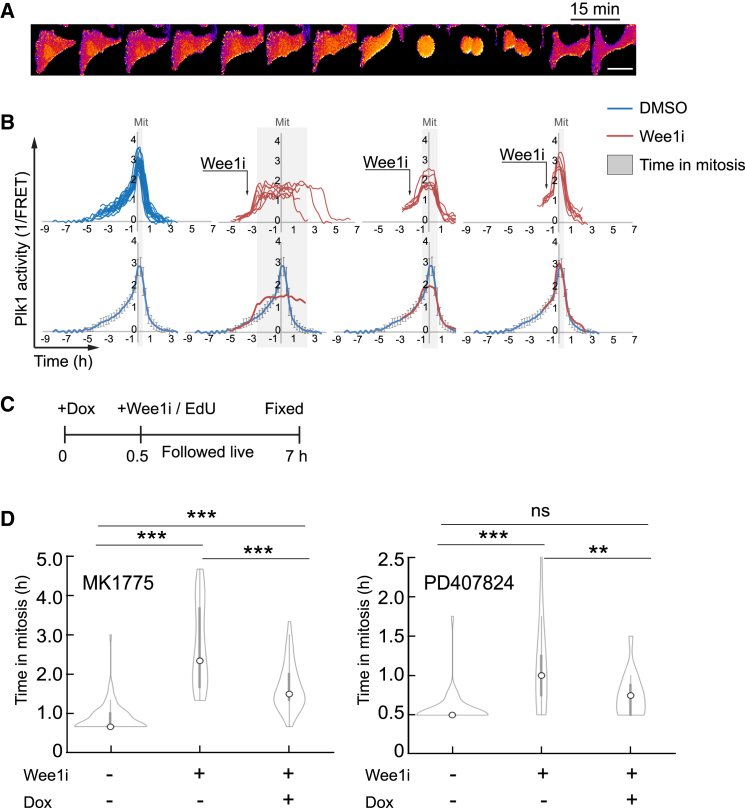
Decoupled Cdk1 and Plk1 activities contribute to prolonged mitosis after Wee1 inhibition (A) Time-lapse imaging of U2OS cells expressing Plk1-FRET probe. Lighter colors indicate phosphorylated probe. Time lapse 15 min. Scale bar 20 μm (B) Dynamics of Plk1 activity in single U2OS cells expressing Plk1-FRET probe (top) and average with standard deviation (bottom). Blue line in bottom panel corresponds to average values of cells treated with DMSO only, and red line to average for cells treated with Wee1i (MK1775). Arrows represent the time when MK1775 (1 μM) was added. The gray area indicates the average duration of mitosis. 19 DMSO- and 21 Wee1i-treated cells are shown. Data are representative of 4 independent experiments. (C) Schematic of the setup for (D). (D) Mitotic duration of U2TR-Plk1-T210D cells treated with DMSO (Ctrl), 1 μM MK1775, or 3 μM PD407824 (Wee1i) and Doxycycline (Dox) as indicated. Cells that entered mitosis between 80 and 120 min after Wee1i addition were followed to ensure comparable populations and to exclude cells that were in late G2 phase upon Wee1i addition. Cells were monitored after fixation, and only 5-ethynyl-2′-deoxyuridine (EdU)-negative cells (indicating that they were not in S phase upon inhibitor addition) were included in the analysis. Left graph, 58–87 cells per condition are showed. Right graph, 31–45 cells per condition are showed. Data are representative of 7 (left graph) and 3 (right graph) independent repeats. ∗∗∗*p* < 0.001, ∗∗*p* < 0.01, using ANOVA. Interquartile range and median values are indicated within violin plots.

We next sought to test whether low Plk1 activity in mitosis after Wee1i addition in G2 phase contributes to the long duration of mitosis. We therefore used U2OS cells expressing a fluorescence resonance energy transfer (FRET)-based probe that monitors Plk1 target phosphorylation in single cells (Figure 6A). We monitored FRET while adding Wee1 inhibitor and followed cells through mitosis. Based on the FRET signal of control cells, we estimated the time remaining to mitosis should no inhibitor has been added (Figure 6B, left). Similar to the model, we find that cells entering mitosis after Wee1 inhibitor addition in early G2 phase were entering mitosis with low Plk1 activity (Figure 6B, second left). In contrast, when Wee1 inhibitors were added in late G2 phase, we detect no difference in mitotic Plk1 activity compared to control cells (Figure 6B, right). Thus, the duration of mitosis inversely correlates with the level of Plk1 activity during mitotic entry (Figure 6B, gray rectangles).

To test whether deficient Plk1 activation after Wee1i addition in G2 phase contributes to prolonged mitosis, we sought to increase Plk1 activity after Wee1i was added. To this end, we used U2TR-Plk1-T210D cells, in which a constitutively active form of Plk1 can by expressed by addition of doxycycline. Induction of expression of Plk1 T210D requires both transcription and translation. We added doxycycline 30 min before Wee1i both to allow expression when Wee1i is added and to minimize Plk1 T210D expression before Wee1 inhibition (Figure 6C). As expected, the mitotic duration increased after treating G2 cells with Wee1i. This increase was partially rescued by expression of Plk1 T210D, suggesting that part of the mitotic delay after Wee1 inhibition in G2 phase depends on a lack of active Plk1 (Figure 6D).

## Discussion

“In G2 phase, a cell prepares for mitosis” is a common statement during undergraduate education, but how this preparation is controlled is less clear. Here, we assembled a data-driven ODE model to capture main logic components of interphase, including a feedforward loop that initiates mitotic kinase activation in early G2 phase and feedback loops that ultimately lead to mitotic entry. We combine the model with experiments and conclude that gradual Cdk1 activation through G2 phase both ensures production of mitotic proteins and coordinates activation of mitotic kinases.

It is now one decade since we reported that Cdk1 and Plk1 are activated upon completion of DNA replication, but the reason for a gradual activation initiated several hours before mitosis has been enigmatic.^7^ Full Cdk1 activation results in mitotic entry, but, before such an activity is reached, a gradual activation takes place throughout G2 phase. We find that the Cdk1-dependent feedback loops that involve transcription of factors that enhance Cdk1 activity also promote production of many mitotic factors that do not directly regulate Cdk1. We suggest that these feedback loops ensure the presence of proteins that are required for mitotic progression when mitosis is triggered.

The majority of Cyclin B-Cdk1 resides in the cytoplasm during G2 phase, raising the question how Cdk1 can regulate transcription. We note that Cdk1-mediated phosphorylation in early G2 phase is first detected in the nucleus and can be reduced by both Cyclin A2 and Cyclin B1 small interfering RNA.^7^ Both Cyclin A2 and Cyclin B1 continuously shuttle between the nucleus and the cytoplasm.^31^^,^^32^ Cyclin A2 partially relocates to the cytoplasm after the S/G2 transition, and both nuclear and cytoplasmic Cyclin A2-Cdk1 can be observed in G2 phase.^33^ Interestingly, active Cyclin B1-Cdk1 has been reported to translocate to the nucleus, possibly providing an explanation for how a seemingly cytoplasmic complex can regulate transcription.^34^^,^^35^

Whereas the transcriptional regulation ensures the presence of proteins, their activities are regulated by posttranslational modifications. We find that the gradual activation of Cdk1 ensures that both Cdk1 and Plk1 are active upon mitotic entry. Thus, a gradual activation throughout G2 phase allows coordinated activities of Cdk1 and Plk1 when mitosis occurs. We find that this coordinated accumulation of mitotic factors and Cdk1 and Plk1 activities are disrupted when Cdk1-dependent feedback loops are shortcut by addition of Wee1 inhibitors. The resulting increased mitotic duration is likely also affected by slower activation kinetics as previously reported.^36^

Wee1 inhibitors are used in clinical trials with the rationales that Wee1 inhibition can overrule a G2 checkpoint and that cells are forced to mitosis prematurely, which leads to mitotic delays and mitotic catastrophe.^37^ The inhibitor AZD1775 has been suggested to also target Plk1, although whether Plk1 inhibition occurs at clinically relevant concentrations has been questioned.^38^^,^^39^ Here, we find that the magnitude of a mitotic delay depends on the time point in G2 phase when Wee1 inhibitors are added. When Wee1 inhibitors were added close to mitosis, only minimal effects on mitotic duration were observed. This is in stark contrast to Plk1 inhibitors, for which a mitotic delay is observed even if added close to mitosis.^30^ Further, measurement of Plk1 activity in single cells using a FRET-based reporter shows no decrease in Plk1 activity upon addition of a Wee1 inhibitor. Rather, as predicted by modeling, cells retained similar Plk1 activity as they had in G2 phase at the time Wee1 inhibitor was added (Figures 5 and 6).

Our findings show a mechanistic rationale for why both Wee1 inhibitor treatment and genetic abrogation of Wee1 show synergistic effects with Plk1 inhibition.^39^ Further, they are consistent with synergistic effects between Aurora A, the upstream kinase activating Plk1, and Wee1 inhibition in head and neck cancer models.^40^ To date, 79 clinical trials using Wee1 inhibitors are reported, and a number of suggested biomarkers for treatment efficiency are available.^41^ Our finding that expression of active Plk1 partially can rescue prolonged mitosis after Wee1 inhibition opens avenues for further investigations on whether combined Wee1 and Plk1 inhibition would be beneficial and whether Plk1 activity can predict Wee1 inhibitor treatment efficiency.

### Limitations of the study

Several experiments in this study use manual tracking of live cells, which limits the number of cells that are analyzed. Further, it cannot be excluded that chemical inhibitors used have non-specific effects. The modeling is based on a selection of key interactions and does therefore not contain all known regulators of the proteins studied. The model does further not encompass the complexity of a cell, including spatial aspects. It should therefore be seen as a minimal theoretical model, and the validity of predictions needs to be tested experimentally. The experiments in this study are performed in 2D cultures of two transformed (U2OS and HeLa) and one untransformed (RPE1) human cell line. Whether they are valid in other cell types, including primary cells, or in a 3D situation, is not clear from this study.

## Resource availability

### Lead contact

Requests for further information and resources should be directed to and will be fulfilled by the lead contact, Arne Lindqvist (arne.lindqvist@ki.se).

### Materials availability

This study did not generate new unique reagents.

### Data and code availability

• Assessed gene expression data and analysis report of RNA sequencing data are present at Mendeley data and are accessible at https://data.mendeley.com/datasets/npt9r48zcz/1 (https://doi.org/10.17632/npt9r48zcz.1). The RNA sequencing analysis report is further available as data S2. Raw read data are not available.
• The code for the cell-cycle model is present as a systems biology markup language (SBML) version at Mendeley data and is accessible at https://data.mendeley.com/datasets/npt9r48zcz/1 (https://doi.org/10.17632/npt9r48zcz.1).
• All data reported in this paper will be shared by the lead contact upon request.
• Any additional information needed to reanalyze the data reported in this paper is available from the lead author upon request.

## Acknowledgments

This study was funded by grants from the Swedish Research Council and the Swedish Cancer Society to A.L. The authors acknowledge support from 10.13039/501100009252Science for Life Laboratory, the Knut and Alice Wallenberg Foundation, the National Genomics Infrastructure funded by the Swedish Research Council, and 10.13039/501100015701Uppsala Multidisciplinary Center for Advanced Computational Science for assistance with massively parallel sequencing and access to the UPPMAX computational infrastructure. The authors thank Rene Medema, Libor Macurek, and Helfrid Hochegger for reagents.

## Author contributions

Conceptualization, A.L. and K.A.; methodology, K.A.; investigation, K.A. and Z.H.; writing – original draft, A.L.; writing – review and editing, A.L., K.A., and Z.H.; funding acquisition, A.L.; supervision, A.L.

## Declaration of interests

The authors declare no competing interests.

## STAR★Methods

### Key resources table

**Table:** 

REAGENT or RESOURCE	SOURCE	IDENTIFIER
**Antibodies**		

Mouse monoclonal anti- Cyclin B1 (V152)	Cell Signaling Technology	Cat# 4135, RRID:AB_2233956
Mouse monoclonal anti- PLK1 (F-8)	Santa Cruz Biotechnology	Cat# sc-17783, RRID:AB_628157
Rabbit polyclonal anti- Cyclin A2	Atlas antibodies	Cat# HPA020626, RRID:AB_1847376
Rabbit polyclonal anti- Phospho-TCTP (Ser46)	Cell Signaling Technology	Cat# 5251, RRID:AB_10547143
Rabbit monoclonal anti- Aurora A/AIK (1G4)	Cell Signaling Technology	Cat# 4718, RRID:AB_2061482
Rabbit polyclonal anti- Aurora B/AIM1	Cell Signaling Technology	Cat# 3094, RRID:AB_10695307
Rabbit polyclonal anti- Phospho-Lamin A/C (Ser22)	Cell Signaling Technology	Cat# 2026, RRID:AB_2136155
Rabbit monoclonal anti- p21 Waf1/Cip1 (12D1)	Cell Signaling Technology	Cat# 2947, RRID:AB_823586
Rabbit polyclonal anti- MASTL	Thermo Fisher Scientific	Cat# A302-190A, RRID:AB_1659819
Rabbit monoclonal anti- Phospho-Histone H3 (Ser10)	Cell Signaling Technology	Cat# 3377, RRID:AB_1549592
Mouse monoclonal anti- Phospho-Histone H3 (Ser 10)	Cell Signaling Technology	Cat# 9706, RRID:AB_331748
Alexa Fluor 488-Goat anti-Rabbit	Molecular Probes	Cat# A-11008, RRID:AB_143165
Alexa Fluor 555-Goat anti-Mouse	Molecular Probes	Cat# A-21422, RRID:AB_141822
		

**Chemicals, peptides, and recombinant proteins**		

RO3306	CalBiochem	#217699
1NMPP1	CalBiochem	#529581
BI2536	Selleck Chemicals	#S1109
NU6140	CalBiochem	#238804
MK1775 (Synonyms: AZD1775, Adavosertib)	Selleckchem	#S1525
PD0166285	Selleckchem	#S8148
PD407824	Sigma	PZ0111
Doxycycline	Sigma	D1822
Thymidine	Sigma	T9250-1G
Cycloheximide	Sigma	C4859
EdU (5-ethynyl-2′-deoxyuridine)	Sigma	900584
Copper(II) sulfate pentahydrate	Sigma	C3036
Ascorbic acid	Sigma	A1300000
Azid Flourophore	Invitrogen	#A10277
		

**Deposited data**		

Assessed gene expression data and analysis report of RNA sequencing data. Raw read data is not available.	This study	https://data.mendeley.com/datasets/npt9r48zcz/1 (https://doi.org/10.17632/npt9r48zcz.1)
		

**Experimental models: Cell lines**		

U2OS CyclinB1-YFP cells	Akopyan et al., 2014^7^	N/A
hTERT RPE1 Cyclin B1-YFP cells	Akopyan et al., 2014^7^	N/A
U2OS cells	Macůrek et al., 2008^42^	N/A
U2OS Plk1-FRET cells	Macůrek et al., 2008^42^	N/A
U2TR - Plk1-T210D cells	Macůrek et al., 2008^42^	N/A
U2OS-Cdk1as cells	Rata et al., 2018^21^	N/A
U2OS-Cdk reporter, PCNA-RFP cells	Lemmens et al., 2018^5^	N/A
		

**Software and algorithms**		

ImageJ	NIH	https://imagej.nih.gov/ij
Python	Python	https://www.python.org
DESeq2 packagein R	R	https://genepattern.github.io/DESeq2
Gene Ontology enRIchment anaLysis and visuaLizAtion	Gorilla	https://cbl-gorilla.cs.technion.ac.il
Copasi 4.44, build 295	Copasi	https://copasi.org/
ODE for cell cycle modeling	This study	https://data.mendeley.com/datasets/npt9r48zcz/1 (https://doi.org/10.17632/npt9r48zcz.1)

### Experimental model and study participants details

#### Cells and cell culture

U2OS CyclinB1-YFP, and hTERT RPE1 Cyclin B1-YFP cells were from.^7^ U2OS, U2OS Plk1-FRET and U2TR - Plk1-T210D cells were from.^42^ U2OS-Cdk1as cells were from.^21^

U2OS and HeLa cells were cultured in DMEM plus GlutaMAX (Invitrogen) supplemented with either 6% (for U2OS and U2OS CyclinB1-YFP) or 10% (for HeLa, U2OS-Cdk1as, U2OS Plk1-FRET and U2TR - Plk1-T210D), heat-inactivated FBS (HyClone) and 1% Penicillin-Streptomycin (HyClone). hTERT RPE1 Cyclin B1-YFP were cultured in DMEM/F12 plus GlutaMAX (Invitrogen) supplemented with 10% heat-inactivated FBS (HyClone) and 1% Penicillin-Streptomycin (HyClone). All cells were maintained in an incubator with controlled conditions at 37°C and 5% CO_2_. No formal authentication was performed for cell type. Cells were tested for Mycoplasma contamination.

For live-cell imaging experiments, the medium of the cells was changed 24 h prior to imaging to CO_2_-independent medium (Leibovitz 15; Invitrogen) supplemented with 6% or 10% heat-inactivated FBS and 1% Penicillin-Streptomycin (HyClone).

### Method details

#### Cell synchronization

Cells were synchronized for 20 h with 2.5 mM thymidine (Sigma-Aldrich) and released into fresh medium for 12 h, followed by 2.5 mM thymidine for an additional 14 h.

#### Live-cell imaging

A DeltaVision Spectris Imaging System with a 20× air objective (NA 0.75), a Leica DMI6000 Imaging System with a 40× air objective (NA 0.85) or 20× air objective (NA 0.40) or an IncuCyte S3 Live-Cell Analysis System with a 20× air objective (NA 0.45) were used to follow the cells live. Images were acquired with an interval of 15–30 min.

The images were analyzed using ImageJ (https://imagej.nih.gov/ij/) or Python.

Imaging and quantification of the CFP/YFP emission ratio FRET cells was performed as described in.^43^

Fibronectin-coated micropatterns (CYTOO) were used as described in.^7^

#### Fixed-cell imaging

Cells were seeded in a 96-well imaging plate (BD Falcon) 24 h prior fixation. The fixation (3.7% formaldehyde (Sigma Aldrich) for 5 min) was followed by permeabilization for 2 min in ice-cold methanol. Fixed cells were incubated in blocking solution: TBS-T (TBS with 0.1% Tween 20) supplemented with 2% bovine albumin serum for 1 h at room temperature. Then cells were incubated in in blocking solution with primary antibodies overnight at 4°C, washed 3 times in TBS-T and incubated with secondary antibodies and DAPI for 1h at room temperature. Samples were washed 3 times in TBS-T and stored in TBS or PBS.

EdU-Click chemistry was performed by incubation in Tris (pH 6.8) – 100mM, CuSO4 – 1mM, Ascorbic acid– 10mM and Azid Flourophore (#A10277, Invitrogen) – 3μM for 30 min at room temperature.

Images were acquired using an ImageXpress microscope with 20× objective (NA 0.45) at room temperature. Image analysis and background subtraction was performed as in.^44^

#### Flow cytometry

Mitotic cells were identified as cells having high pH3 level and 4N DNA content using a BD FACSCanto II flow cytometer. Cells were fixed using 3.7% formaldehyde followed by methanol. For DNA content analysis, cells were treated with 100 μg/mL RNase A and stained with 1 μg/mL DAPI. For PLK1 activity or level analysis, cells were washed using FACS buffer (DPBS solution, 2 mM EDTA, 0.5% BSA, 0.1% Tween 20) when fixation and staining. Data were analyzed using FlowJo software. Cell doublets were excluded for all analyses.

#### RNA sequencing

4.5 h after double thymidine block release HeLa cells were treated for 2 h either with Cdk1 inhibitor (RO3306) or DMSO. RNA isolation, sequencing, quality control and mapping of reads were performed by the National Genomics Infrastructure at SciLifeLab. Briefly, strand-specific TruSeq RNA libraries were prepared after poly-A selection and sequenced using an Illumina HiSeq sequencer. Reads were mapped using Tophat, RPKM/FPKM values were calculated using Cufflinks and read counts were calculated using HTSeq.

Data analysis was performed using the DESeq2 package (https://genepattern.github.io/DESeq2) in R.

Gene ontology analysis was performed using Gene Ontology enRIchment anaLysis and visuaLizAtion tool – Gorilla (https://cbl-gorilla.cs.technion.ac.il)

#### Modeling

The mathematical model used a modified form of Michaelis–Menten rate law based on the “total” quasi-steady state approximation, as described in.^45^ A detailed description of all equations and parameters can be found in Data S1.

Solving the equations and parameter estimation were performed using Copasi 4.44, build 295 (https://copasi.org/)

### Quantification and statistical analysis

Quantification of image data was performed using ImageJ or Python as described in.^43^^,^^44^

Student’s t-Test or an ANOVA test followed by Tukey’s HSD post-hoc test were used to calculate *p*-values using Python or R. A significance level of *p* < 0.05 was set. Statistical details of the experiments, including the statistical tests used, number of cells, number of experimental repetitions, definition of center, and dispersion and precision measures are indicated in the figure legends.
