# Supplementary material for: Preparation for mitosis requires gradual CDK1 activation
Source: iScience. 2025 Mar 25;28(5):112292. doi: 10.1016/j.isci.2025.112292 (PMC12008674; doi:10.1016/j.isci.2025.112292)
Supplement: Document S1. Figures S1–S5 and Data S1 and S2 [file mmc1.pdf]

iScience, Volume *28*

Supplemental information

Preparation for mitosis requires gradual CDK1 activation

Karen Akopyan, Zhiyu Hao, and Arne Lindqvist

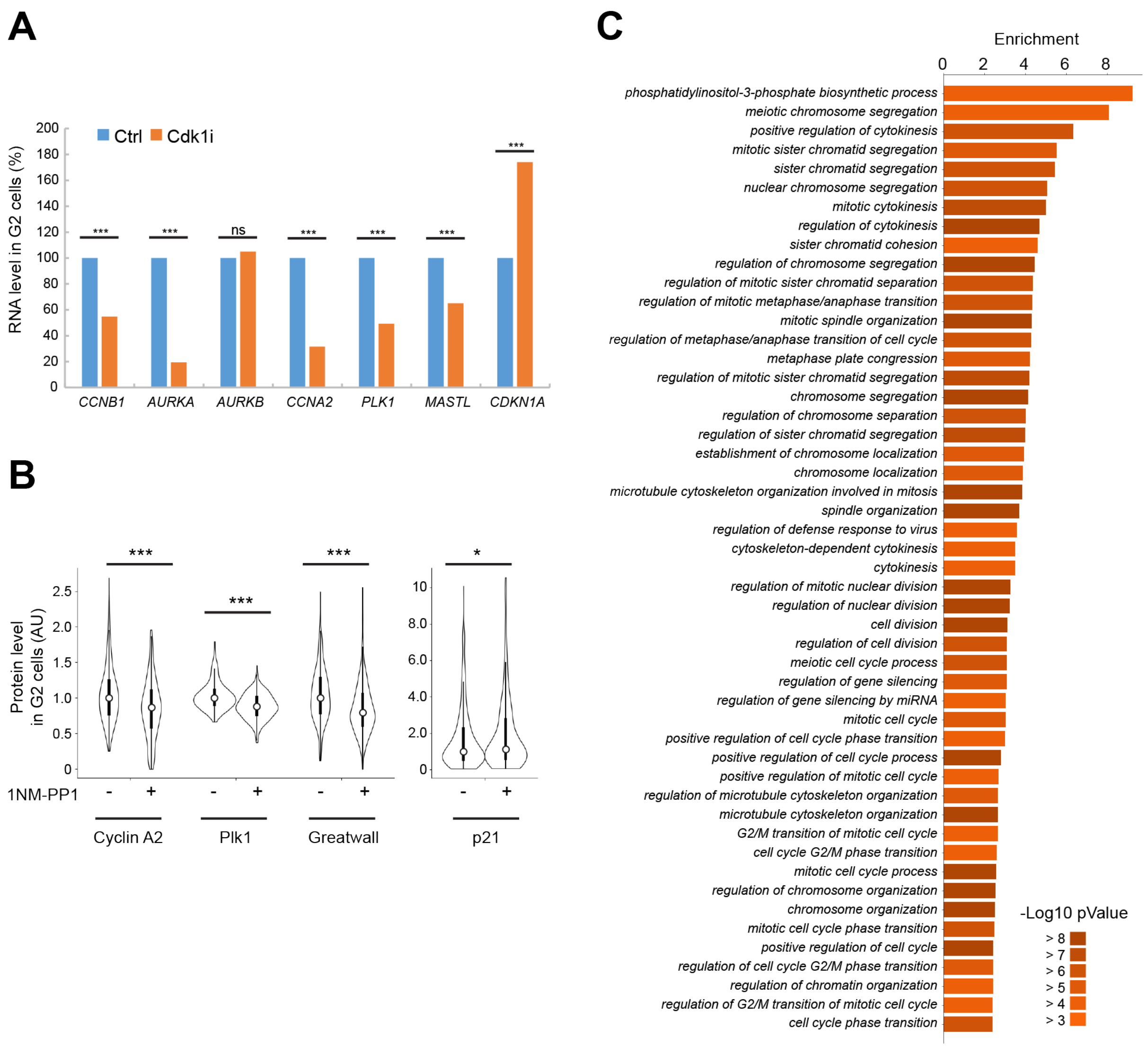

Figure S1. Cdk1 regulates accumulation of key cell cycle regulators in G2 phase, related to Figure 2. (A) RNA levels relative to control for indicated genes. Based on data for figure 2C (***P < 0.001, using Student's t-Test). **(B)** Quantification of Cyclin A2, Plk1, Greatwall and p21 immunofluorescence in 4N U2OS-Cdk1as cells after 2h treatment with 1NM-PP1. Data shows 173 – 471 cells per condition and is representative of two independent experiments. ***P < 0.001, *P < 0.05, using Student's t-Test. **(C)** Enrichment of GO terms. Expansion of data showed in figure 2F.

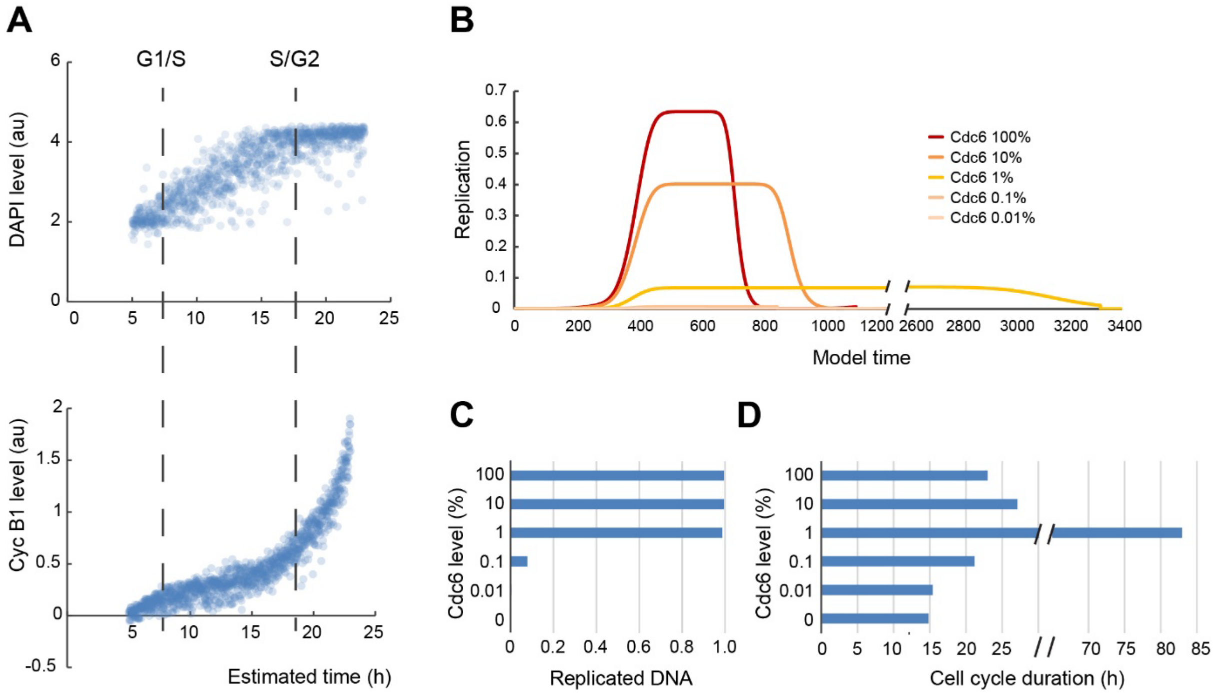

Figure S2, related to Figure 3. (A) Example of G1/S and S/G2 border determination based on DAPI staining after ordering cells based on increasing DAPI and Cyclin B1 content. For estimation of time and comparison to time-lapse microscopy, see (Akopyan et al., 2014). **(B)** Simulation of replication dynamics with indicated levels of Cdc6 in % of unperturbed model. (**C**) Total amount of DNA replicated before mitosis with indicated levels of Cdc6 in % of unperturbed model. **(D)** Cell cycle duration with indicated levels of Cdc6 in % of unperturbed model.

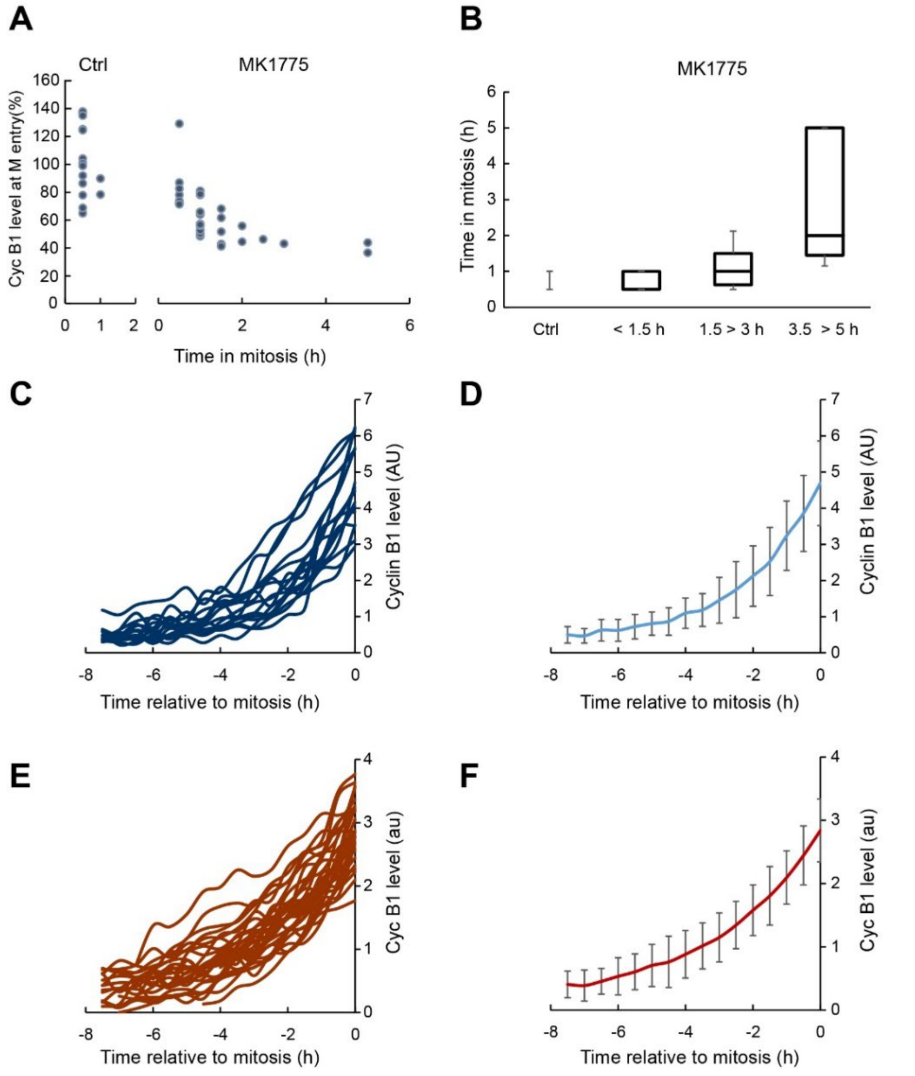

Figure S3, related to Figure 4. (A, B) Duration of mitosis after adding Wee1i to hTERT RPE1 Cyclin B1-YFP cells. RPE1 Cyclin B1-YFP cells were monitored by time-lapse microscopy upon addition of 1µM MK1775 (30 cells treated with MK1775 and 16 control cells are shown, data is representative of more than three independent experiments). **A**, duration of mitosis (x-axis) is plotted versus Cyclin B1-YFP level at mitotic entry (y- axis) **B**, duration of mitosis (y-axis) is plotted versus estimated time before mitosis should Wee1 inhibitors have not been added (x-axis).

(C, D) Quantification and trendline used to estimate position within G2 phase for B. RPE1 Cyclin B1-YFP cells were monitored by live cell microscopy. Plots show single cell traces (**C**) and average with standard deviation (**D**) of YFP fluorescence after *in silico* synchronization in mitosis. Cell cycle position for individual cells in B was estimated by comparison to the average trend line (15 individual cells representative of 3 independent experiments).

(E, F) Quantification and trendline used to estimate position within G2 phase for Figure 4E. U2OS Cyclin B1-YFP cells were monitored by live cell microscopy. Plots show single cell traces (**E**) and average (**F**) of YFP fluorescence after *in silico* synchronization in mitosis. Cell cycle position for individual cells in Figure 4E was estimated by comparison to the average trend line (30 individual cells representative of 3 independent experiments).

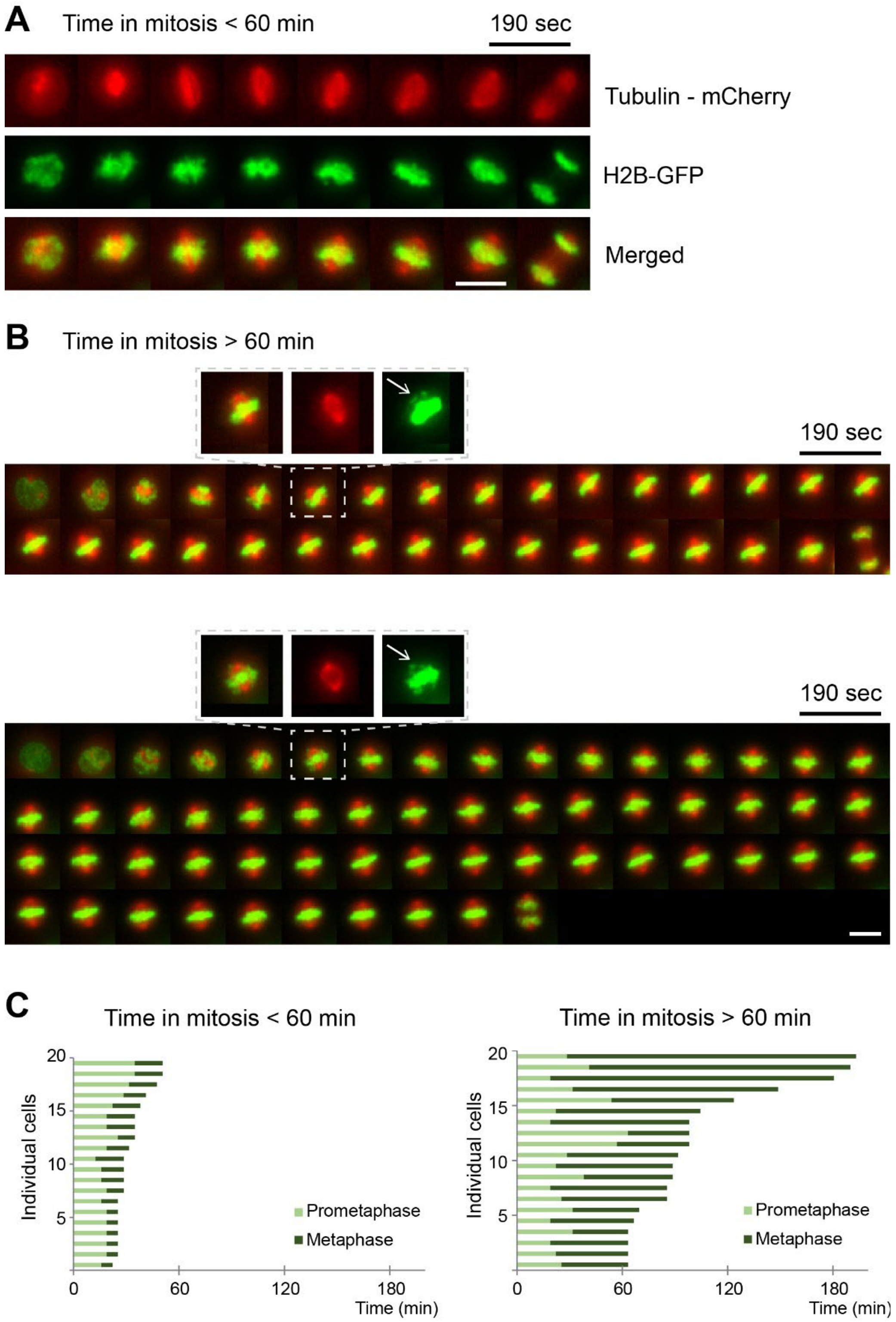

Figure S4. Mitosis after Wee1i addition in G2 phase, related to Figure 4.
(A, B) Representative time-lapse microscopy images of U2OS cells expressing H2B-GFP and Tub-mCherry after Wee1 inhibition (MK1775, 1μM). Arrows point to chromosomes not aligned to the metaphase plate. Scale bar 20 μm. **(C)** Quantification of prometaphase and metaphase. Prometaphase is scored as all chromosomes not aligned to the metaphase plate, metaphase is scored as all chromosomes aligned to the metaphase plate (40 individual cells from 3 independent experiments). No apparent lagging chromosomes or anaphase defects were observed.

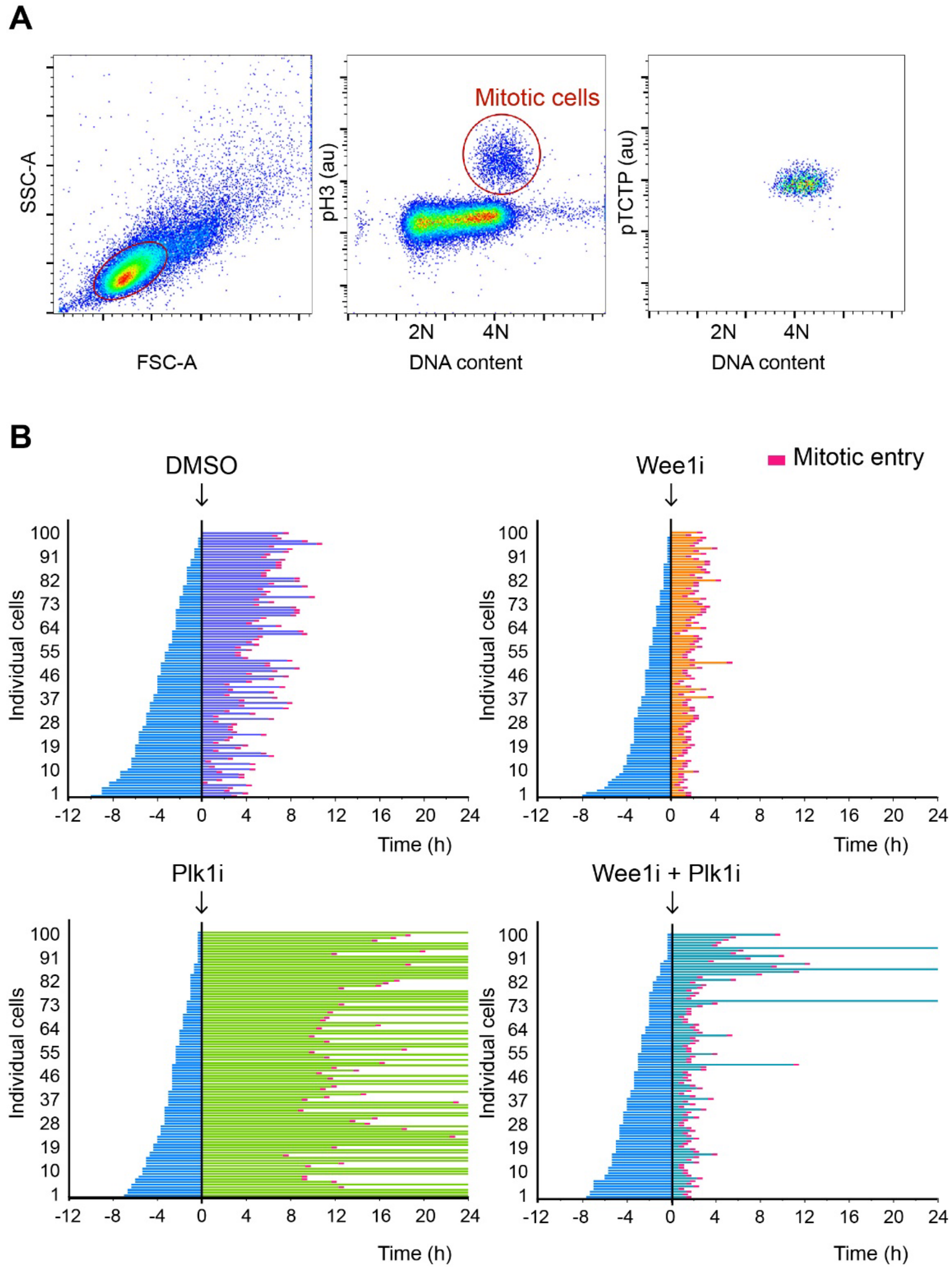

Figure S5, related to Figure 5. (A) Flow cytometry gating strategy for figure 5C. Cells were first gated based on FSC/SSC to exclude cell aggregates. Mitotic cells were identified by high pH3 signal intensity and 4N DNA content based on DAPI staining. Within the mitotic cell population, PLK1 or pTCTP expression levels were quantified and compared across different experimental groups, with two replicates per group. **(B)** Mitotic entry after Wee1 and Plk1 inhibitor addition in G2 phase. Tracking individual cell cycle fates in U2OS CDK reporter, PCNA-RFP cells treated with PLK1 inhibitor (BI-2356, 100nM) and/or Wee1 inhibitor (MK1775, 1μM). The onset of G2 phase, marked by the disappearance of PCNA foci, is indicated as start of a blue line. Indicated inhibitors were added at timepoint 0. Cells entering G2 phase before time 0 were analyzed for G2-to-mitosis duration under different treatments. 100 cells per condition were analyzed in two independent experiments.

Supplementary Data 1: cell cycle model

The model is based on a slightly modified form of Michaelis–Menten rate law based on the "total" quasisteady state approximation [S1].

$$\frac{dP}{dt} = \frac{k_{cat}}{2}\left[Km + E_T + \hat{S} - \sqrt{\left(Km + E_T + \hat{S}\right)^2 - 4E_T\hat{S}}\right]$$

where $\hat{S} = S - P$, and S, E, and P are the time-dependent concentrations of substrate (S), enzyme (E), and product (P), respectively.

To avoid overparameterization all Michaelis constants (*Km*) in the model are set to one and the level of dynamic variables is relative to the *Km* values.

All equations in the model are assigned into 3 groups corresponding to cell cycle phases in which they are considered most relevant. The following labels were added to protein names to indicate: "T" – total level, "P" – phosphorylated, "A" – active, "I" – inactive.

For some proteins the total level is defined as constant = 1.

Due to the lack of compartmentalization in the model, the $CycB_A$ component encompasses both nuclear and cytoplasmic Cdk1 activity, primarily involving Cyclin B-Cdk1, but also including Cyclin A-Cdk1.

For simplicity, the mitotic phase is excluded from the model. Instead, once the cell enters mitosis ([$CycB_A$] >1), all corresponding parameters are reset to their initial levels.

Time: the total cell cycle time is set to 920 where the G2 starts approximately at model time 740. The experimental data we used for tuning indicated that the cell cycle was approximately 23 hours (Figures 3B and S3). This translates to a ratio of 40 model time units equaling 1 hour.

There are two parameters in the model that direct protein synthesis:

1. *Slow constitutive synthesis* is a constant (specified as E2F in Fig. 3). The constant is different for every protein.
2. *Active synthesis* denotes regulated expression. For simplicity, factors are given as the species FoxM (specified as FoxM/NF-Y in Fig. 3).

G2 phase

FoxM

The level of active FoxM [$FoxM_A$] is regulated by cell cycle kinases Cdk1, Cdk2 [S2-S5] and Plk1 [S6-S8].

$$\begin{aligned}\frac{d[FoxM_A]}{dt} = &\frac{1}{2}k_{cycbfoxm}\left(Km + [CycB_A] + [FoxM_I] - \sqrt{(Km + [CycB_A] + [FoxM_I])^2 - 4[CycB_A][FoxM_I]}\right)\\ &+ \frac{1}{2}k_{cycafoxm}\left(Km + [CycA_A] + [FoxM_I] - \sqrt{(Km + [CycA_A] + [FoxM_I])^2 - 4[CycA_A][FoxM_I]}\right)\\ &+ \frac{1}{2}k_{cycefoxm}\left(Km + [CycE_A] + [FoxM_I] - \sqrt{(Km + [CycE_A] + [FoxM_I])^2 - 4[CycE_A][FoxM_I]}\right)\\ &+ \frac{1}{2}k_{plk1foxm}\left(Km + [Plk1_A] + [FoxM_I] - \sqrt{(Km + [Plk1_A] + [FoxM_I])^2 - 4[Plk1_A][FoxM_I]}\right)\\ &- \frac{1}{2}k_{pp2foxm}\left(Km + [PP2_A] + [FoxM_A] - \sqrt{(Km + [PP2_A] + [FoxM_A])^2 - 4[PP2_A][FoxM_A]}\right)\end{aligned}$$

$$[FoxM_I] = [FoxM_T] - [FoxM_A]$$

CycBCdk1

The term CycBCdk1 denotes both Cyclin B-Cdk1 and Cyclin A-Cdk1 complexes. The total level of CycBCdk1 is described by the following equation:

$$\frac{d[CycB_T]}{dt} = k_{scycb} + V_{scycbfoxm} - V_{dcycb} \cdot [CycB_T]$$

Where k_{scycb} is a constant corresponding to slow constitutive synthesis, $V_{scycbfoxm}$ is a rate function describing the regulation of *Active synthesis*. And V_{dcycb} function describes the degradation. Here, we assume that all Cyclin B binds immediately to Cdk1 forming Cyclin B-Cdk1 complexes.

The level of active Cyclin B-Cdk1 [$CycB_A$] is regulated by the balance between Cdc25C phosphatase and Wee1/Myt1 kinases. The inhibitory phosphorylation on Thr 14 and Tyr 15 by Wee1/Myt1 [S9, S10] turns Cdk1 to an inactive phosphorylated form [$CycB_I$] and Cdc25 turns it back to an active form by removing those phosphogroups [S11-S13].

$$\begin{aligned}\frac{d[CycB_A]}{dt} = &\frac{1}{2}k_{cdc25cycb}\left(Km + [Cdc25C_A] + [CycB_I] - \sqrt{(Km + [Cdc25C_A] + [CycB_I])^2 - 4[Cdc25C_A][CycB_I]}\right)\\ &+ \frac{1}{2}k_{cdc25abcycb}\left(Km + [Cdc25AB_A] + [CycB_I] - \sqrt{(Km + [Cdc25AB_A] + [CycB_I])^2 - 4[Cdc25AB_A][CycB_I]}\right)\\ &- \frac{1}{2}k_{wee1cycb}\left(Km + [Wee1_A] + [CycB_A] - \sqrt{(Km + [Wee1_A] + [CycB_A])^2 - 4[Wee1_A][CycB_A]}\right)\end{aligned}$$

The level of inactive Cyclin B-Cdk1 [$CycB_I$] is defined by:

$$[CycB_I] = [CycB_T] - [CycB_A]$$

CycACdk2

The term CycACdk2 denotes Cyclin A-Cdk2 complexes. The total level of CycACdk2 is given by:

$$\frac{d[CycA_T]}{dt} = k_{scyca} + V_{scycafoxm} - V_{dcyca} \cdot [CycA_T]$$

Where k_{scyca} is a constant corresponding to synthesis, $V_{scycafoxm}$ is a rate function describing the regulation of *Active synthesis* and V_{dcyca} function describes the degradation of Cyclin A.

Inhibition/Association of CycA by p27:

$$\frac{d[CycAp27]}{dt} = k_{assa} \cdot [CycA_A] \cdot ([p27_T] - [CycAp27] - [CycEp27]) - \left(k_{disa} + V_{dp27} + V_{dcyca}\right) \cdot [CycAp27]$$

The level of active CycA-Cdk2 is regulated by Cdc25AB and Wee1:

$$\frac{d[CycA_A]}{dt} = \frac{1}{2}k_{cdc25cyca}\left(Km + [Cdc25AB_A] + [CycA_I] - \sqrt{(Km + [Cdc25AB_A] + [CycA_I])^2 - 4[Cdc25AB_A][CycA_I]}\right) - \frac{1}{2}k_{wee1cyca}\left(Km + [Wee1_A] + [CycA_A] - \sqrt{(Km + [Wee1_A] + [CycA_A])^2 - 4[Wee1_A][CycA_A]}\right)$$

The level of inactive CycA-Cdk2 is defined by:

$$[CycA_I] = [CycA_T] - [CycAp27] - [CycA_A]$$

Plk1

The total level of Plk1 is defined by:

$$\frac{d[Plk1_T]}{dt} = k_{splk1} + V_{splk1foxm} - V_{dplk1} \cdot [Plk1_T]$$

k_{splk1} is a constant corresponding to S*low constitutive synthesis*, $V_{splk1foxm}$ describes the regulation of *Active synthesis*, and V_{dplk1} function describes the degradation.

The level of active Plk1 is regulated by Aurora A kinase with cofactor Bora [AurBora] [S14, S15] and Chk1 [S16]:

$$\frac{d[Plk1_A]}{dt} = \frac{1}{2}k_{aurboraplk1}\left(Km + [AurBora] + [Plk1_I] - \sqrt{(Km + [AurBora] + [Plk1_I])^2 - 4[AurBora][Plk1_I]}\right) - \frac{1}{2}k_{chk1plk1}\left(Km + [Chk1_A] + [Plk1_A] - \sqrt{(Km + [Chk1_A] + [Plk1_A])^2 - 4[Chk1_A][Plk1_A]}\right)$$

The level of inactive Plk1 is defined by:

$$[Plk1_I] = [Plk1_T] - [Plk1_A]$$

Aurora A

The total level of Aurora A is defined by:

$$\frac{d[AurA_T]}{dt} = k_{saura} + V_{saurafoxm} - V_{daur} \cdot [AurA_T]$$

k_{saura} is a constant corresponding to S*low constitutive synthesis*, $V_{saurafoxm}$ describes the regulation of *Active synthesis*, and V_{daur} function describes the degradation.

For simplicity we do not involve an Aurora activation – inactivation path. We assume that Aurora A is always active:

$$[AurA] = [AurA_T] - [AurBora]$$

PP2

We use [PP2] as the collective value of all phosphatases opposing Cdk1 [S17].

Cdk1 is involved in inactivation of PP2 (by Greatwall) [S18]. For activation of PP2 we use the constant (k_{pp2pp2})

$$\frac{d[PP2_I]}{dt} = \frac{1}{2}k_{cycbpp2}\left(Km + [CycB_A] + [PP2_A] - \sqrt{(Km + [CycB_A] + [PP2_A])^2 - 4[CycB_A][PP2_A]}\right) - \frac{1}{2}k_{pp2pp2}\left(Km + [PP2_I] - \sqrt{(Km + [PP2_I])^2 - 4[PP2_I]}\right)$$

The level of active PP2 is defined by:

$$[PP2_A] = [PP2_T] - [PP2_I]$$

Bora

In our model Bora is present in 2 forms: not phosphorylated by Cdk1 [Bora] and phosphorylated by Cdk1 [$Bora_P$] [S19, S20].

Plk1 is involved in the degradation of Bora [Bora] while Cdk1 and Cdk2 can phosphorylate Bora which leads to stabilization [S21].

$$\frac{d[Bora]}{dt} = k_{sbora} + V_{sborafoxm} + \frac{1}{2}k_{pp2bora}\left(Km + [PP2_A] + [Bora_P] - \sqrt{(Km + [PP2_A] + [Bora_P])^2 - 4[PP2_A][Bora_P]}\right)$$
$$- \frac{1}{2}k_{cycbbora}\left(Km + [CycB_A] + [Bora] - \sqrt{(Km + [CycB_A] + [Bora])^2 - 4[CycB_A][Bora]}\right) - V_{dbora} \cdot [Bora]$$
$$- V_{dboraplk1} \cdot [Bora]$$

$$\frac{d[Bora_P]}{dt} = \frac{1}{2}k_{cycbbora}\left(Km + [CycB_A] + [Bora] - \sqrt{(Km + [CycB_A] + [Bora])^2 - 4[CycB_A][Bora]}\right)$$
$$+ \frac{1}{2}k_{cycabora}\left(Km + [CycA_A] + [Bora] - \sqrt{(Km + [CycA_A] + [Bora])^2 - 4[CycA_A][Bora]}\right)$$
$$- \frac{1}{2}k_{pp2bora}\left(Km + [PP2_A] + [Bora_P] - \sqrt{(Km + [PP2_A] + [Bora_P])^2 - 4[PP2_A][Bora_P]}\right) - V_{dbora} \cdot [Bora_P]$$

Aurora A - Bora

Aurora A form a complex with Bora phosphorylated by Cdk1 [AurBora] [S22].

$$\frac{d[AurBora]}{dt} = k_{assb} \cdot [AurA] \cdot [Bora_P] - (k_{disb} + V_{daur} + V_{dbora}) \cdot [AurBora]$$

Cdc25C

The total level of Cdc25C is defined as a constant.

For activation of Cdc25C we use the following equation:

$$\frac{d[Cdc25C_A]}{dt} = \frac{1}{2}k_{cycbcdc25}\left(Km + [CycB_A] + [Cdc25C_I] - \sqrt{(Km + [CycB_A] + [Cdc25C_I])^2 - 4[CycB_A][Cdc25C_I]}\right)$$
$$+ \frac{1}{2}k_{plk1cdc25}\left(Km + [Plk1_A] + [Cdc25C_I] - \sqrt{(Km + [Plk1_A] + [Cdc25C_I])^2 - 4[Plk1_A][Cdc25C_I]}\right)$$
$$+ \frac{1}{2}k_{aurcdc25}\left(Km + [AurA] + [Cdc25C_I] - \sqrt{(Km + [AurA] + [Cdc25C_I])^2 - 4[AurA][Cdc25C_I]}\right)$$
$$- \frac{1}{2}k_{pp2cdc25}\left(Km + [PP2_A] + [Cdc25C_A] - \sqrt{(Km + [PP2_A] + [Cdc25C_A])^2 - 4[PP2_A][Cdc25C_A]}\right)$$
$$- \frac{1}{2}k_{chk1cdc25}\left(Km + [Chk1_A] + [Cdc25C_A]\right.$$
$$\left.- \sqrt{(Km + [Chk1_A] + [Cdc25C_A])^2 - 4[Chk1_A][Cdc25C_A]}\right)$$

Cdk1 as well as Plk1 and Aurora A promote activation [S23-S25], and PP2 and Chk1 promote inactivation of Cdc25C [S25].

The level of inactive Cdc25C is defined by:

$$[Cdc25C_I] = [Cdc25C_T] - [Cdc25C_A]$$

Wee1

The total level of Wee1 is defined as a constant.

Inactivation of Wee1 by Cdk and Plk1 [S26, S27]:

$$\frac{d[Wee1_I]}{dt} = \frac{1}{2}k_{cycbwee1}\left(Km + [CycB_A] + [Wee1_A] - \sqrt{(Km + [CycB_A] + [Wee1_A])^2 - 4[CycB_A][Wee1_A]}\right)$$
$$+ \frac{1}{2}k_{plk1wee1}\left(Km + [Plk1_A] + [Wee1_A] - \sqrt{(Km + [Plk1_A] + [Wee1_A])^2 - 4[Plk1_A][Wee1_A]}\right)$$
$$+ \frac{1}{2}k_{cycawee1}\left(Km + [CycA_A] + [Wee1_A] - \sqrt{(Km + [CycA_A] + [Wee1_A])^2 - 4[CycA_A][Wee1_A]}\right)$$
$$+ \frac{1}{2}k_{cycewee1}\left(Km + [CycE_A] + [Wee1_A] - \sqrt{(Km + [CycE_A] + [Wee1_A])^2 - 4[CycE_A][Wee1_A]}\right)$$
$$- \frac{1}{2}k_{pp2wee1}\left(Km + [PP2_A] + [Wee1_I] - \sqrt{(Km + [PP2_A] + [Wee1_I])^2 - 4[PP2_A][Wee1_I]}\right)$$

The level of active Wee1 is defined by:

$$[Wee1_A] = [Wee1_T] - [Wee1_I]$$

S-phase

CycECdk2

$$\frac{d[CycE_T]}{dt} = k_{scyce} - V_{dcyce} \cdot [CycE_T]$$

$$\frac{d[CycEp27]}{dt} = k_{asse} \cdot [CycE_A] \cdot ([p27_T] - [CycAp27] - [CycEp27]) - \left(k_{dise} + V_{dp27} + V_{dcyce}\right) \cdot [CycEp27]$$

$$\frac{d[CycE_A]}{dt} = \frac{1}{2}k_{cdc25cyce}\left(Km + [Cdc25AB_A] + [CycE_I] - \sqrt{(Km + [Cdc25AB_A] + [CycE_I])^2 - 4[Cdc25AB_A][CycE_I]}\right)$$
$$- \frac{1}{2}k_{wee1cyce}\left(Km + [Wee1_A] + [CycE_A] - \sqrt{(Km + [Wee1_A] + [CycE_I])^2 - 4[Wee1_A][CycE_A]}\right)$$

$$[CycE_I] = [CycE_T] - [CycEp27] - [CycE_A]$$

Cdc25AB

For simplicity we combined two isoforms of Cdc25 (Cdc25A and Cdc25B) [Cdc25AB].

The total level of Cdc25AB is defined as a constant.

For activation of Cdc25AB we used the following equation:

$$
\begin{aligned}
\frac{d[Cdc25AB_A]}{dt} = & \frac{1}{2}k_{cycecdc25a}\left(Km + [CycE_A] + [Cdc25AB_I] - \sqrt{(Km + [CycE_A] + [Cdc25AB_I])^2 - 4[CycE_A][Cdc25AB_I]}\right) \\
& + \frac{1}{2}k_{cycacdc25a}\left(Km + [CycA_A] + [Cdc25AB_I] - \sqrt{(Km + [CycA_A] + [Cdc25AB_I])^2 - 4[CycA_A][Cdc25AB_I]}\right) \\
& + \frac{1}{2}k_{plk1cdc25}\left(Km + [Plk1_A] + [Cdc25AB_I] - \sqrt{(Km + [Plk1_A] + [Cdc25AB_I])^2 - 4[Plk1_A][Cdc25AB_I]}\right) \\
& + \frac{1}{2}k_{aurcdc25}\left(Km + [AurA] + [Cdc25AB_I] - \sqrt{(Km + [AurA] + [Cdc25AB_I])^2 - 4[AurA][Cdc25AB_I]}\right) \\
& - \frac{1}{2}k_{pp2cdc25}\left(Km + [PP2_A] + [Cdc25AB_A] - \sqrt{(Km + [PP2_A] + [Cdc25AB_A])^2 - 4[PP2_A][Cdc25AB_A]}\right) \\
& - \frac{1}{2}k_{chk1cdc25ab}\left(Km + [Chk1_A] + [Cdc25AB_A] - \sqrt{(Km + [Chk1_A] + [Cdc25AB_A])^2 - 4[Chk1_A][Cdc25AB_A]}\right)
\end{aligned}
$$

Cdk2, Plk1 and Aurora A activate Cdc25AB, whereas PP2 and Chk1 inactivate Cdc25AB [S25, S28-S30].

The level of inactive Cdc25AB is defined by:

$$[Cdc25AB_I] = [Cdc25AB_T] - [Cdc25AB_A]$$

ATR

The total level of ATR is defined as a constant.

For activation of ATR we used the following equation:

$$
\begin{aligned}
\frac{d[Atr_A]}{dt} = & \frac{1}{2}k_{replatr}\left(Km + [Origin_F :: Replication] + [Atr_I] - \sqrt{(Km + [COrigin_F :: Replication] + [Atr_I])^2 - 4[Origin_F :: Replication][Atr_I]}\right) \\
& - \frac{1}{2}k_{autatr}\left(Km + [Atr_A] - \sqrt{(Km + [Atr_A])^2 - 4[Atr_A]}\right)
\end{aligned}
$$

ATR is activated by Replication [S31, S32]. For simplicity we used a constant k_{autatr} to denote inactivation of ATR.

The level of inactive ATR is defined by:

$$[Atr_I] = [Atr_T] - [Atr_A]$$

Chk1

The total level of ATR is defined as a constant.

ATR-dependent Chk1 activation is described by:

$$
\begin{aligned}
\frac{d[Chk1_A]}{dt} = & \frac{1}{2}k_{atrchk1}\left(Km + [Atr_A] + [Chk1_I] - \sqrt{(Km + [Atr_A] + [Chk1_I])^2 - 4[Atr_A][Chk1_I]}\right) \\
& - \frac{1}{2}k_{plk1chk1}\left(Km + [Plk1_A] + [Chk1_A] - \sqrt{(Km + [Plk1_A] + [Chk1_A])^2 - 4[Plk1_A][Chk1_A]}\right) \\
& - \frac{1}{2}k_{autchk1}\left(Km + [Chk1_A] - \sqrt{(Km + [Chk1_A])^2 - 4[Chk1_A]}\right)
\end{aligned}
$$

Plk1 inactivates Chk1 [S33-S35] by inducing degradation of Claspin, which is required for activation of Chk1.

The level of inactive Chk1 is defined by:

$$[Chk1_I] = [Chk1_T] - [Chk1_A]$$

Cdc6

The total level of Cdc6 is defined as a constant.

Cdk2 dependent inactivation of Cdc6 [S36-S38]:

$$\frac{d[Cdc6_I]}{dt} = \frac{1}{2}k_{cycecdc6}\left(Km + [CycE_A] + [Cdc6_A] - \sqrt{(Km + [CycE_A] + [Cdc6_A])^2 - 4[CycE_A][Cdc6_A]}\right) + \frac{1}{2}k_{cycacdc6}\left(Km + [CycA_A] + [Cdc6_A] - \sqrt{(Km + [CycA_A] + [Cdc6_A])^2 - 4[CycA_A][Cdc6_A]}\right)$$

The level of active Cdc6 is defined by:

$$[Cdc6_A] = [Cdc6_T] - [Cdc6_I]$$

Cdc7

The total level of Cdc7 is described as:

$$\frac{d[Cdc7_T]}{dt} = k_{scdc7} - V_{dcdc7} \cdot [Cdc7_T]$$

The k_{scdc7} is a constant corresponding to *Slow constitutive synthesis*, whereas V_{dcdc7} function describes the degradation.

Cdk2 dependent Cdc7 activation [36–38] is described by:

$$\frac{d[Cdc7_A]}{dt} = \frac{1}{2}k_{cycecdc7}\left(Km + [CycE_A] + [Cdc7_I] - \sqrt{(Km + [CycE_A] + [Cdc7_I])^2 - 4[CycE_A][Cdc7_I]}\right) + \frac{1}{2}k_{cycacdc7}\left(Km + [CycA_A] + [Cdc7_I] - \sqrt{(Km + [CycA_A] + [Cdc7_I])^2 - 4[CycA_A][Cdc7_I]}\right)$$

The level of inactive Cdc7 is defined by:

$$[Cdc7_I] = [Cdc7_T] - [Cdc7_A]$$

DNA replication

Is described by the following equations:

The first step, loading of Orc, Cdc6, and MCM proteins [Cdc6] onto DNA is described as Origin licensing [$Origin_L$] [S36-S40]:

$$\frac{d[Origin_L]}{dt} = k_{assl} \cdot [Cdc6_A] \cdot [Origin] - \frac{1}{2}k_{assf}\left(Km + [Cdc7_A] + [Origin_L] - \sqrt{(Km + [Cdc7_A] + [Origin_L])^2 - 4[Cdc7_A][Origin_L]}\right) - V_{repl} \cdot [Origin_L]$$

The second step is described as Origin firing by Cdc7 [S36-S38, S40]:

$$\frac{d[Origin_F :: Replication]}{dt} = \frac{1}{2}k_{assf}\left(Km + [Cdc7_A] + [Origin_L] - \sqrt{(Km + [Cdc7_A] + [Origin_L])^2 - 4[Cdc7_A][Origin_L]}\right)$$

$$- [Origin_F :: Replication] \cdot [Origin_D]^{kodr}$$

$$\frac{d[Origin_D]}{dt} = V_{repl} \cdot ([Origin_T] - [Origin_D])$$

$$[Origin] = [Origin_T] - [Origin_L] - [Origin_F :: Replication]$$

G1-phase, taken from [S41].

$$\frac{d[Skp2]}{dt} = k_{sskp2} - \left(k_{dskp2} + k_{dskp2c1} \cdot [Cdh1]\right) \cdot [Skp2]$$

$$\frac{d[Cdh1]}{dt} = k_{acdh1} \cdot (Cdh1_T - [EmiC] - [Cdh1]) - V_{icdh1} \cdot [Cdh1] - k_{asec} \cdot [Cdh1] \cdot ([Emi1_T] - [EmiC]) + (k_{diec} + k_{demi1}) \cdot ([Cdh1dp] - [Cdh1])$$

$$\frac{d[EmiC]}{dt} = k_{asec} \cdot (Cdh1_T - [EmiC]) \cdot ([Emi1_T] - [EmiC]) - (k_{diec} + k_{demi1}) \cdot [EmiC]$$

$$\frac{d[Cdh1dp]}{dt} = k_{acdh1} - ([Cdh1_T] - [Cdh1dp]) - V_{icdh1} \cdot [Cdh1dp]$$

$$\frac{d[p27_T]}{dt} = k_{s27} - V_{dp27} \cdot [p27_T]$$

Global Quantities (Type: assignment)

$$V_{icdh1} = k_{icdh1e} \cdot [CycE_A] + k_{icdh1a} \cdot [CycA_A]$$

$$V_{dcyce} = k_{dcyce} + k_{dcycee} \cdot [CycE_A] + k_{dcycea} \cdot [CycA_A]$$

$$V_{dcyca} = k_{dcyca} + k_{dcycac1} \cdot [Cdh1]$$

$$V_{dp27} = (k_{d27e} \cdot [CycE_A] + k_{d27a} \cdot [CycA_A]) \cdot [Skp2] + k_{d27}$$

$$V_{dcycb} = k_{dcycb} + k_{dcycbc1} \cdot [Cdh1]$$

$$V_{dplk1} = k_{dplk1} + k_{dplk1c1} \cdot [Cdh1]$$

$$V_{daur} = k_{daur} + k_{daurc1} \cdot [Cdh1]$$

$$V_{dbora} = k_{dbora} + k_{dborac1} \cdot [Cdh1]$$

$$V_{dboraplk1} = k_{dboraplk1} \cdot [Plk1_A]$$

$$V_{scycbfoxm} = k_{scycbfoxm} \cdot [FoxM_A]$$

$$V_{saurafoxm} = k_{saurafoxm} \cdot [FoxM_A]$$

$$V_{splk1foxm} = k_{splk1foxm} \cdot [FoxM_A]$$

$$V_{scycafoxm} = k_{scycafoxm} \cdot [FoxM_A]$$

$$V_{sborafoxm} = k_{sborafoxm} \cdot [FoxM_A]$$

$$V_{dcdc7} = k_{dcdc7} + k_{dcdc7c1} \cdot [Cdh1]$$

$$V_{repl} = k_{repl} \cdot [Origin_F :: Replication]$$

Global Quantities (Type: fixed)

#	Name	Value		#	Name	Value		#	Name	Value
1	ksskp2	0.004		35	kdaurc1	0.75090424		69	kchk1wee1	200
2	kdskp2	0.002		36	kcycbcdc25	0.101625007		70	kchk1cdc25	10
3	kdskp2c1	0.2		37	kpp2cdc25	0.09027315		71	kreplatr	0.5
4	Cdh1T	1		38	kplk1cdc25	2.09183084		72	katrchk1	0.3
5	kacdh1	0.02		39	kaurcdc25	0.1		73	kautatr	0.25
6	kicdh1e	0.07		40	kwee1cycb	44.72172096		74	kautchk1	0.4
7	kicdh1a	0.2		41	kcdc25cycb	8.6289313		75	kcdc25cyca	50
8	kasec	2		42	kautplk1	0.05		76	kcdc25cyce	50
9	kdiec	0.02		43	kaurboraplk1	0.08		77	kwee1cyca	0.5
10	ksemi1	0.003		44	kcycbpp2	0.155115167		78	kwee1cyce	14
11	kdemi1	0.001		45	kpp2pp2	0.05		79	kcycacdc25a	0.1
12	ks27	0.008		46	kcycbwee1	0.64369118		80	kcycecdc25a	0.05
13	kd27	0.004		47	kpp2wee1	0.658411743		81	kcycabora	0.055959715
14	kd27e	2		48	kplk1wee1	0.735261119		82	kplk1chk1	0.5
15	kd27a	2		49	kcycbprobe	2		83	kchk1plk1	25
16	kscyca	0.00253		50	kpp2probe	1		84	kscdc7	1
17	kdcyca	0.002		51	kassb	0.550645521		85	kdcdc7	0.001
18	kdcycac1	0.4		52	kdisb	0.02		86	kdcdc7c1	100
19	kassa	1		53	kdbora	0.072778314		87	kcdc25abcycb	1.5
20	kdisa	0.02		54	kdborac1	0.567370959		88	kchk1cdc25ab	0.1
21	kscyce	0.005		55	ksbora	0.002748359		89	kcycawee1	0.05
22	kdcyce	0.001		56	kpp2bora	0.096448508		90	kcycewee1	0.8
23	kdcycee	0.0001		57	kcycbbora	3.445732371		91	kodr	400
24	kdcycea	0.06		58	kdboraplk1	0.011541844		92	kcycbfoxm	1.8
25	kasse	1		59	kcycacdc6	5		93	kcycafoxm	0.09
26	kdise	0.02		60	kcycecdc6	0.1		94	kcycefoxm	0.15
27	kscycb	0.0002		61	kcycacdc7	0.005		95	kplk1foxm	0.01
28	kdcycb	0.002		62	kcycecdc7	0.001		96	kpp2foxm	1
29	kdcycbc1	0.35		63	kassl	0.1		97	kscycbfoxm	0.03
30	ksplk1	0.003736186		64	kdisl	0		98	ksplk1foxm	0.09
31	kdplk1	0.000229651		65	kassf	1		99	kscycafoxm	0.005
32	kdplk1c1	0.109550453		66	kdisf	0		100	ksborafoxm	0.0053
33	ksaura	0.002134667		67	krepl	0.0235		101	Km	1
34	kdaur	0.01		68	ksaurafoxm	0.049820691				

Supplemental references

1. Kim, J.K., and Tyson, J.J. (2020). Misuse of the Michaelis-Menten rate law for protein interaction networks and its remedy. PLoS Comput. Biol. *16*, e1008258. https://doi.org/10.1371/journal.pcbi.1008258.

2. Laoukili, J., Alvarez, M., Meijer, L.A.T., Stahl, M., Mohammed, S., Kleij, L., Heck, A.J.R., and Medema, R.H. (2008). Activation of FoxM1 during G2 requires cyclin A/Cdk-dependent relief of autorepression by the FoxM1 N-terminal domain. Mol. Cell. Biol. *28*, 3076–3087. https://doi.org/10.1128/MCB.01710-07.

3. Lüscher-Firzlaff, J.M., Lilischkis, R., and Lüscher, B. (2006). Regulation of the transcription factor FOXM1c by Cyclin E/CDK2. FEBS Lett. *580*, 1716–1722. https://doi.org/10.1016/j.febslet.2006.02.021.

4. Major, M.L., Lepe, R., and Costa, R.H. (2004). Forkhead box M1B transcriptional activity requires binding of Cdk-cyclin complexes for phosphorylation-dependent recruitment of p300/CBP coactivators. Mol. Cell. Biol. *24*, 2649–2661. https://doi.org/10.1128/mcb.24.7.2649-2661.2004.

5. Saldivar, J.C., Hamperl, S., Bocek, M.J., Chung, M., Bass, T.E., Cisneros-Soberanis, F., Samejima, K., Xie, L., Paulson, J.R., Earnshaw, W.C., et al. (2018). An intrinsic S/G2 checkpoint enforced by ATR. Science *361*, 806–810. https://doi.org/10.1126/science.aap9346.

6. Fu, Z., Malureanu, L., Huang, J., Wang, W., Li, H., van Deursen, J.M., Tindall, D.J., and Chen, J. (2008). Plk1-dependent phosphorylation of FoxM1 regulates a transcriptional programme required for mitotic progression. Nat. Cell Biol. *10*, 1076–1082. https://doi.org/10.1038/ncb1767.

7. Zhang, J., Yuan, C., Wu, J., Elsayed, Z., and Fu, Z. (2015). Polo-like kinase 1-mediated phosphorylation of Forkhead box protein M1b antagonizes its SUMOylation and facilitates its mitotic function. J. Biol. Chem. *290*, 3708–3719. https://doi.org/10.1074/jbc.M114.634386.

8. Murakami, H., Aiba, H., Nakanishi, M., and Murakami-Tonami, Y. (2010). Regulation of yeast forkhead transcription factors and FoxM1 by cyclin-dependent and polo-like kinases. Cell Cycle *9*, 3253–3262. https://doi.org/10.4161/cc.9.16.12599.

9. Fattaey, A., and Booher, R.N. (1997). Myt1: a Wee1-type kinase that phosphorylates Cdc2 on residue Thr14. In Progress in Cell Cycle Research Progress in Cell Cycle Research., L. Meijer, S. Guidet, and M. Philippe, eds. (Springer US), pp. 233–240. https://doi.org/10.1007/978-1-4615-5371-7_18.

10. Liu, F., Stanton, J.J., Wu, Z., and Piwnica-Worms, H. (1997). The human Myt1 kinase preferentially phosphorylates Cdc2 on threonine 14 and localizes to the endoplasmic reticulum and Golgi complex. Mol. Cell. Biol. *17*, 571–583. https://doi.org/10.1128/mcb.17.2.571.

11. Hoffmann, I., Clarke, P.R., Marcote, M.J., Karsenti, E., and Draetta, G. (1993). Phosphorylation and activation of human cdc25-C by cdc2--cyclin B and its involvement in the self-amplification of MPF at mitosis. EMBO J. *12*, 53–63.

12. Kumagai, A., and Dunphy, W.G. (1992). Regulation of the cdc25 protein during the cell cycle in Xenopus extracts. Cell *70*, 139–151. https://doi.org/10.1016/0092-8674(92)90540-S.

13. Kumagai, A., and Dunphy, W.G. (1991). The cdc25 protein controls tyrosine dephosphorylation of the cdc2 protein in a cell-free system. Cell *64*, 903–914. https://doi.org/10.1016/0092-8674(91)90315-p.

14. Macůrek, L., Lindqvist, A., Lim, D., Lampson, M.A., Klompmaker, R., Freire, R., Clouin, C., Taylor, S.S., Yaffe, M.B., and Medema, R.H. (2008). Polo-like kinase-1 is activated by aurora A to promote checkpoint recovery. Nature *455*, 119–123. https://doi.org/10.1038/nature07185.

15. Seki, A., Coppinger, J.A., Jang, C.-Y., Yates, J.R., and Fang, G. (2008). Bora and Aurora A Cooperatively Activate Plk1 and Control the Entry into Mitosis. Science *320*, 1655–1658. https://doi.org/10.1126/science.1157425.

16. Bruinsma, W., Aprelia, M., García-Santisteban, I., Kool, J., Xu, Y.J., and Medema, R.H. (2017). Inhibition of Polo-like kinase 1 during the DNA damage response is mediated through loss of Aurora A recruitment by Bora. Oncogene *36*, 1840–1848. https://doi.org/10.1038/onc.2016.347.

17. Grallert, A., Boke, E., Hagting, A., Hodgson, B., Connolly, Y., Griffiths, J.R., Smith, D.L., Pines, J., and Hagan, I.M. (2015). A PP1-PP2A phosphatase relay controls mitotic progression. Nature *517*, 94–98. https://doi.org/10.1038/nature14019.

18. Okumura, E., Morita, A., Wakai, M., Mochida, S., Hara, M., and Kishimoto, T. (2014). Cyclin B-Cdk1 inhibits protein phosphatase PP2A-B55 via a Greatwall kinase-independent mechanism. J. Cell Biol. *204*, 881–889. https://doi.org/10.1083/jcb.201307160.

19. Thomas, Y., Cirillo, L., Panbianco, C., Martino, L., Tavernier, N., Schwager, F., Van Hove, L., Joly, N., Santamaria, A., Pintard, L., et al. (2016). Cdk1 Phosphorylates SPAT-1/Bora to Promote Plk1 Activation in C. elegans and Human Cells. Cell Rep. *15*, 510–518. https://doi.org/10.1016/j.celrep.2016.03.049.

20. Vigneron, S., Sundermann, L., Labbé, J.-C., Pintard, L., Radulescu, O., Castro, A., and Lorca, T. (2018). Cyclin A-cdk1-Dependent Phosphorylation of Bora Is the Triggering Factor Promoting Mitotic Entry. Dev. Cell *45*, 637-650.e7. https://doi.org/10.1016/j.devcel.2018.05.005.

21. Feine, O., Hukasova, E., Bruinsma, W., Freire, R., Fainsod, A., Gannon, J., Mahbubani, H.M., Lindqvist, A., and Brandeis, M. (2014). Phosphorylation-mediated stabilization of Bora in mitosis coordinates Plx1/Plk1 and Cdk1 oscillations. Cell Cycle Georget. Tex *13*, 1727–1736. https://doi.org/10.4161/cc.28630.

22. Parrilla, A., Cirillo, L., Thomas, Y., Gotta, M., Pintard, L., and Santamaria, A. (2016). Mitotic entry: The interplay between Cdk1, Plk1 and Bora. Cell Cycle Georget. Tex *15*, 3177–3182. https://doi.org/10.1080/15384101.2016.1249544.

23. Gheghiani, L., Loew, D., Lombard, B., Mansfeld, J., and Gavet, O. (2017). PLK1 Activation in Late G2 Sets Up Commitment to Mitosis. Cell Rep. *19*, 2060–2073. https://doi.org/10.1016/j.celrep.2017.05.031.

24. Roshak, A.K., Capper, E.A., Imburgia, C., Fornwald, J., Scott, G., and Marshall, L.A. (2000). The human polo-like kinase, PLK, regulates cdc2/cyclin B through phosphorylation and activation of the cdc25C phosphatase. Cell. Signal. *12*, 405–411. https://doi.org/10.1016/S0898-6568(00)00080-2.

25. Sur, S., and Agrawal, D.K. (2016). Phosphatases and kinases regulating CDC25 activity in the cell cycle: clinical implications of CDC25 overexpression and potential treatment strategies. Mol. Cell. Biochem. *416*, 33–46. https://doi.org/10.1007/s11010-016-2693-2.

26. Nakajima, H., Toyoshima-Morimoto, F., Taniguchi, E., and Nishida, E. (2003). Identification of a Consensus Motif for Plk (Polo-like Kinase) Phosphorylation Reveals Myt1 as a Plk1 Substrate*. J. Biol. Chem. *278*, 25277–25280. https://doi.org/10.1074/jbc.C300126200.

27. Watanabe, N., Arai, H., Iwasaki, J., Shiina, M., Ogata, K., Hunter, T., and Osada, H. (2005). Cyclin-dependent kinase (CDK) phosphorylation destabilizes somatic Wee1 via multiple pathways. Proc. Natl. Acad. Sci. *102*, 11663–11668. https://doi.org/10.1073/pnas.0500410102.

28. Lobjois, V., Froment, C., Braud, E., Grimal, F., Burlet-Schiltz, O., Ducommun, B., and Bouche, J.-P. (2011). Study of the docking-dependent PLK1 phosphorylation of the CDC25B phosphatase. Biochem. Biophys. Res. Commun. *410*, 87–90. https://doi.org/10.1016/j.bbrc.2011.05.110.

29. Mailand, N., Falck, J., Lukas, C., Syljuâsen, R.G., Welcker, M., Bartek, J., and Lukas, J. (2000). Rapid destruction of human Cdc25A in response to DNA damage. Science *288*, 1425–1429. https://doi.org/10.1126/science.288.5470.1425.

30. Reinhardt, H.C., Aslanian, A.S., Lees, J.A., and Yaffe, M.B. (2007). p53-deficient cells rely on ATM- and ATR-mediated checkpoint signaling through the p38MAPK/MK2 pathway for survival after DNA damage. Cancer Cell *11*, 175–189. https://doi.org/10.1016/j.ccr.2006.11.024.

31. Friedel, A.M., Pike, B.L., and Gasser, S.M. (2009). ATR/Mec1: coordinating fork stability and repair. Curr. Opin. Cell Biol. *21*, 237–244. https://doi.org/10.1016/j.ceb.2009.01.017.

32. Toledo, L.I., Altmeyer, M., Rask, M.-B., Lukas, C., Larsen, D.H., Povlsen, L.K., Bekker-Jensen, S., Mailand, N., Bartek, J., and Lukas, J. (2013). ATR Prohibits Replication Catastrophe by Preventing Global Exhaustion of RPA. Cell *155*, 1088–1103. https://doi.org/10.1016/j.cell.2013.10.043.

33. Mailand, N., Bekker-Jensen, S., Bartek, J., and Lukas, J. (2006). Destruction of Claspin by SCFβTrCP Restrains Chk1 Activation and Facilitates Recovery from Genotoxic Stress. Mol. Cell *23*, 307–318. https://doi.org/10.1016/j.molcel.2006.06.016.

34. Mamely, I., van Vugt, M.A., Smits, V.A., Semple, J.I., Lemmens, B., Perrakis, A., Medema, R.H., and Freire, R. (2006). Polo-like Kinase-1 Controls Proteasome-Dependent Degradation of Claspin during Checkpoint Recovery. Curr. Biol. *16*, 1950–1955. https://doi.org/10.1016/j.cub.2006.08.026.

35. Peschiaroli, A., Dorrello, N.V., Guardavaccaro, D., Venere, M., Halazonetis, T., Sherman, N.E., and Pagano, M. (2006). SCFβTrCP-Mediated Degradation of Claspin Regulates Recovery from the DNA Replication Checkpoint Response. Mol. Cell *23*, 319–329. https://doi.org/10.1016/j.molcel.2006.06.013.

36. Coulombe, P., Nassar, J., Peiffer, I., Stanojcic, S., Sterkers, Y., Delamarre, A., Bocquet, S., and Méchali, M. (2019). The ORC ubiquitin ligase OBI1 promotes DNA replication origin firing. Nat. Commun. *10*, 1–14. https://doi.org/10.1038/s41467-019-10321-x.

37. Dutta, A., and Bell, S.P. (1997). Initiation of Dna Replication in Eukaryotic Cells. Annu. Rev. Cell Dev. Biol. *13*, 293–332. https://doi.org/10.1146/annurev.cellbio.13.1.293.

38. Michael, W.M., Ott, R., Fanning, E., and Newport, J. (2000). Activation of the DNA Replication Checkpoint Through RNA Synthesis by Primase. Science *289*, 2133–2137. https://doi.org/10.1126/science.289.5487.2133.

39. Bleichert, F. (2019). Mechanisms of replication origin licensing: a structural perspective. Curr. Opin. Struct. Biol. *59*, 195–204. https://doi.org/10.1016/j.sbi.2019.08.007.

40. Lemmens, B., Hegarat, N., Akopyan, K., Sala-Gaston, J., Bartek, J., Hochegger, H., and Lindqvist, A. (2018). DNA Replication Determines Timing of Mitosis by Restricting CDK1 and PLK1 Activation. Mol. Cell *71*, 117-128.e3. https://doi.org/10.1016/j.molcel.2018.05.026.

41. Barr, A.R., Heldt, F.S., Zhang, T., Bakal, C., and Novák, B. (2016). A Dynamical Framework for the All-or-None G1/S Transition. Cell Syst. *2*, 27–37. https://doi.org/10.1016/j.cels.2016.01.001.

RNA-seq analysis report

SciLifeLab Stockholm

2015-09-16

Project name

A.Lindqvist_15_01

UPPNEX project id

b2015212

Access the results on UPPMAX

Please refer to the Sequencing FAQ for detailed instructions on how to log in and access your files. If you have problems to access your data, please contact genomics_support@scilifelab.se. If you have questions regarding UPPNEX, please contact support@uppmax.uu.se.

Specification of RNA-seq analysis delivery

The RNA-seq analysis is delivered to /proj/b2015212/INBOX/A.Lindqvist_15_01/analysis. The delivery consists of:

- **Analysis Report (this document):** Contains reports about: Mapping statistics, Read distribution, Correlation between FPKM values of samples, Gene body covarage, Library complexity and Strand specificity.
- **BAM files:** The alignment files are placed under the alignments folder. We deliver two files per sample, one with and one without duplicates.
- **FPKM files:** The quantification folder contains FPKM values calculated using the Cufflinks program using ENSEMBL annotation of genes and transcripts for each sample. These files also contain the upper and lower limits of the confidence interval for the FPKM estimate. FPKM values are the paired-end equivalent of RPKM (Reads Per Kilobase per Million mapped reads; the standard measure for gene expression in RNA-seq.)
- **fpkm_table.txt:** A single file containing all of the FPKM values per gene and sample (duplicates included). This can be opened in Excel or a regular text processing application.
- **fpkm_table_isoforms.txt:** A single file containing all of the FPKM values per isoform and sample (duplicates included). This can be opened in Excel or a regular text processing application.
- **count_table.txt:** Counts per gene and sample (duplicates included). For analyzing differential expression of genes or transcripts, it may be useful to have the raw read counts (the number of sequences that map to each gene/transcript). These are calculated using the HTSeq software and are collected into the table count_table.txt.

The RNA-seq pipeline step by step

Your samples were analysed as follows:

1. **Mapping:** Reads were mapped with Tophat/2.0.4 to the Human genome assembly, build GRCh37.
2. **Merging:** Bamfiles from samples run on different lanes were merged with samtools.
3. **Sorting and Marking duplicates:** Merged bam files were sorted and duplicates removed using picard-tools/1.29.
4. **Counts for genes:** Gene counts were generated using htseq/0.6.1 on bam files with duplicates included.
5. **FPKMs for genes and transcripts:** FPKMs for genes and transcripts were generated using cufflinks/2.1.1 on bamfiles with duplicates included.
6. **Mapping statistics:** Mapping statistics were calculated from numbers obtained by running bam_stat.py (included in rseqc/2.3.6) on bam files with and without duplicates.
7. **Readdistribution:** read_distribution.py (included in rseqc/2.3.6) was run on bam files with duplicates.
8. **Genebodycovarage:** geneBody_coverage.py (included in rseqc/2.3.6) was run on bam files with duplicates.
9. **Strandedness:** infer_experiment.py (included in rseqc/2.3.6) was run on bam files with duplicates.
10. **Library complexity:** The complexity plot is generated by running preseq/1.0.0 on bam files with duplicates.
11. **Corelation heatmap:** The correlation heatmap is generated using the R-package pheatmap on the FPKM table for genes.

Results

Mapping statistics

Table 1.

Sample	Tot NO Reads	UniqMapped	UniqMapped DuplRem
P2464_101	40863600	86.2%	24.59%
P2464_102	38467941	85.93%	28.12%
P2464_103	67179712	87.36%	19.87%
P2464_104	60806776	87.53%	21.27%
P2464_105	71356550	86.95%	19.45%
P2464_106	44626248	86.09%	24.72%

Tot NO Reads: If paired-end reads, the total number of reads indicates the total number of sequenced paired-end reads. Since a paired-end read is made up of two sequenced fragments (mates), the total number of sequenced 100-bp regions is twice the number shown in this column. If single-end reads, this column reflects the total numer of sequences.

UniqMapped: The number of fragments that are mapped relative to the total number of sequenced fragments.

UniqMapped DuplRem: We remove duplicate reads i.e. reads that map to the same genomic location (if paired-end, duplicates are defined as the paired end reads where both mates map to the same genomic positions). If the sequenced reads/pairs will be used to call sequence variants, it is recommended to use duplicate-removed data to obtain unbiased variant frequency calls. It is not uncommon to observe high rates of read duplication rates in RNA-seq libraries and they may not necessarily be the result of libraries with low complexity and/or over-amplification of libraries. The duplication rates can be gene specific i.e. a small number of genes are being highly expressed and comprise most of the duplicated reads/pairs, hence reflecting the underlying RNA distribution of the sample being investigated. In the case of small number of highly abundant genes consuming majority of the sequenced reads, one might need to sequence the samples deeper to obtain a better distribution of the whole transcriptome. When performing differential expression analyses, it is not recommended to use duplicate-removed reads/pairs. This is due to the fact that removing duplicate reads may effect expression values of transcripts. Since different samples have inherently different RNA distributions with different levels of duplicate reads/pairs for different transcripts, there could be a risk of removing biologically relevant information hence leading to incorrect comparisons.

Read distribution

Table 2 contain information about the extent to which sequences from each sample mapped to different structural parts of genes, like coding exons, untranslated regions, and transcription start sites. The actual number itself is less important than the relative values for the different kinds of regions. For a normal RNA-seq experiment you should have a higher value in the CDS Exon column than in the others, for example. Perhaps the most easily interpretable column is the mRNA column, which gives the percentage of sequences that mapped to ENSEMBL-annotated mRNA (including coding regions and UTRs). While this number is not completely accurate (because ENSEMBL doe not completely describe the transcriptome), it is a useful summary statistic which should be relatively high for an mRNA-seq experiment, typically above 80%.

CDS: Coding sequence exons.

UTR: Untranslated exon region.

TES: Transcription end site Down 1kb.

TSS: Transcription start site Up 1kb.

Intron: Intronic or intergenic regions.

mRNA: Percentage of sequences that mapped to ENSEMBL-annotated mRNA.

Table 2.

Sample	CDS	5'UTR	3'UTR	Intron	TSS	TES	mRNA
P2464_101	375.99	22.01	259.32	1.39	1.92	6.60	93.12%
P2464_102	318.98	22.05	281.83	2.00	2.36	7.48	89.78%
P2464_103	571.10	40.17	495.48	3.15	2.90	11.18	91.02%
P2464_104	519.93	38.61	349.88	4.27	2.29	9.02	87.47%
P2464_105	635.17	42.48	503.90	2.64	2.84	12.01	92.66%
P2464_106	374.86	25.27	324.47	2.22	1.84	8.69	90.34%

Gene body covarage

Read coverage over gene body. To check if reads coverage is uniform and if there is any 5'/3' bias. All transcripts are scaled to 100 nucleotides and the read number is then calculated as the number of reads covering each nucleotide position. Figure 1 shows the average gene body coverage from all samples.

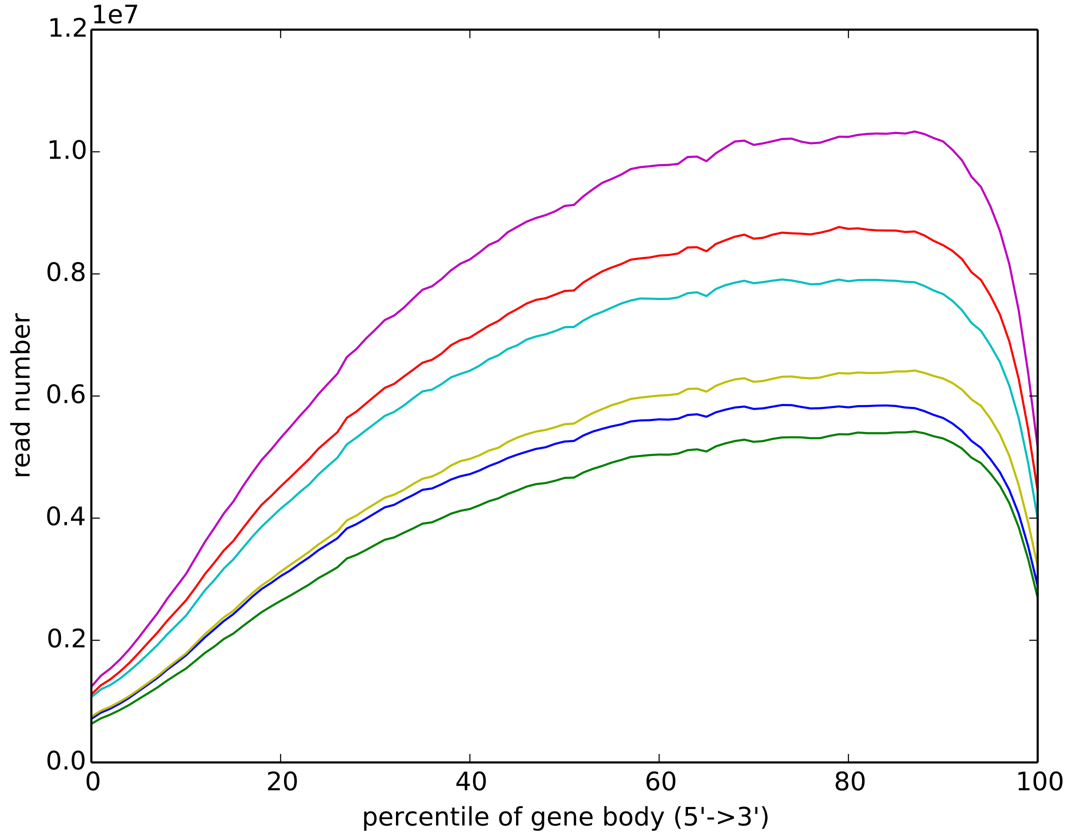

Figure 1.

FPKM heatmap

Figure 2 shows the (Pearson) correlation between FPKM values of samples.

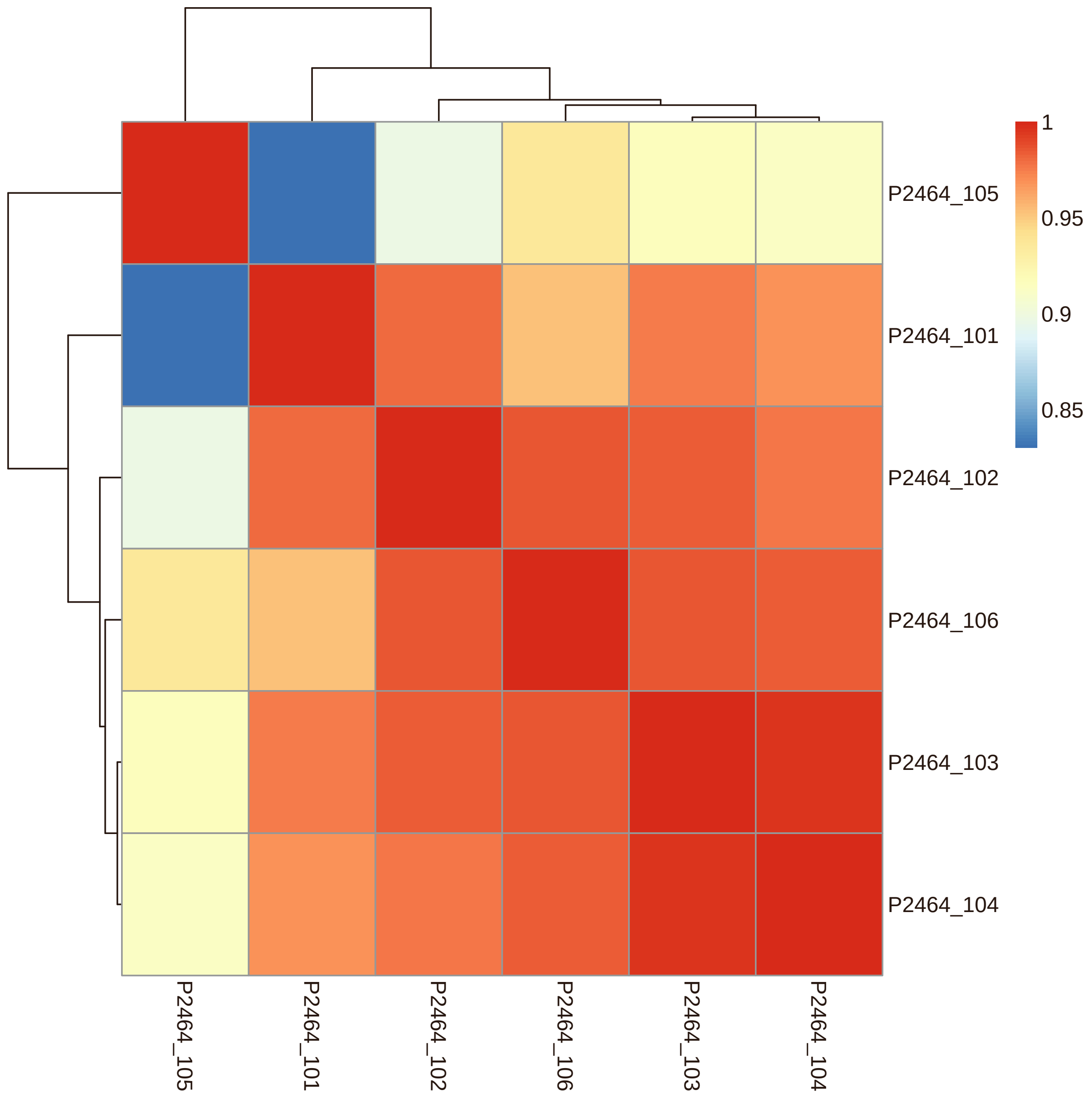

Figure 2.

Library complexity

Library complexity is an important measure when assessing the quality of samples and libraries. It refers to the amount of unique molecules that are present in a sample/library. Complex libraries, i.e. those with high number of unique molecules can be explored by sequencing with high coverage, whereas those with low complexity are probably not worthy of sequencing further or deeper. Figure 3 shows the number of unique molecules detected as a function of number of reads sequenced. It is obtained by extrapolating from the actual sequencing run and can be regarded as a predictor of the complexity of sample/library and assess its potential for further sequencing. For further information, please refer to: http://www.nature.com/nmeth/journal/v10/n4/full/nmeth.2375.html)

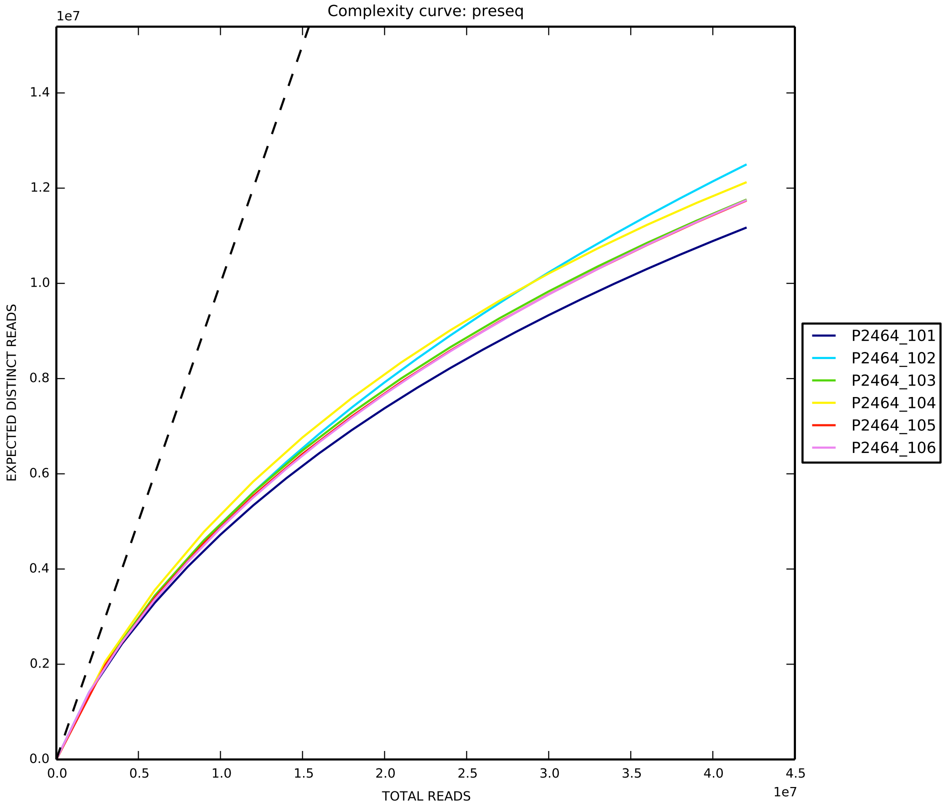

Figure 3.

Quantification of rRNA present in the samples

Presented percentage rRNA per sample. The numbers are calculated as the number of reads mapping to Ensembl genes that have their <source> field set to "rRNA" in the gtf file, divided by the total number of mapped reads.

Table 3.

Sample	rRNA
P2464_101	0.33%
P2464_102	0.34%
P2464_103	0.32%
P2464_104	0.36%
P2464_105	0.23%
P2464_106	0.26%

Percentage of reads/pairs mapped to the expected strand

The script infer_experiment.py from RSeQC package is used to evaluate strand-specificity of the sequencing. Table 4 shows the percentage of read pairs that were detected on the strand the gene is expressed.

Table 4.

Sample	strand-specific reads
P2464_101	98.87%
P2464_102	98.38%
P2464_103	97.89%
P2464_104	96.78%
P2464_105	98.08%
P2464_106	98.12%

Tools and references

Reference genome assembly: Human, GRCh37

http://www.ensembl.org

Ensembl annotation: Release 73

Mapping: Tophat/2.0.4

http://tophat.cbcb.umd.edu/

Duplicate removal: picard-tools/1.29

http://picard.sourceforge.net/command-line-overview.shtml

Read count: htseq/0.6.1

http://www-huber.embl.de/users/anders/HTSeq/doc/overview.html

RPKM/FPKM values: cufflinks/2.1.1

http://cufflinks.cbcb.umd.edu/

Gene body coverage, Read distribution and Strandspecifisity: rseqc/2.3.6

http://rseqc.sourceforge.net/

Library Complexity: preseq/1.0.0

http://smithlabresearch.org/software/preseq/
